# Testing for Mechanistic Interactions in Long-Term Follow-Up Studies

**DOI:** 10.1371/journal.pone.0121638

**Published:** 2015-03-26

**Authors:** Jui-Hsiang Lin, Wen-Chung Lee

**Affiliations:** Research Center for Genes, Environment and Human Health and Institute of Epidemiology and Preventive Medicine, College of Public Health, National Taiwan University, Taipei, Taiwan; Memorial Sloan Kettering Cancer Center, UNITED STATES

## Abstract

In follow-up studies, interactions are often assessed by including a cross-product term in a (multiplicative) Cox model. However, epidemiologists/clinicians often misinterpret a significant multiplicative interaction as a genuine mechanistic interaction. Though indices specific to mechanistic interactions have been proposed, including the ‘relative excess risk due to interaction’ (RERI) and the ‘peril ratio index of synergy based on multiplicativity’ (PRISM), these indices assume no loss to follow up and no competing death in a study. In this paper, the authors propose a novel ‘mechanistic interaction test’ (MIT) for censored data. Monte-Carlo simulation shows that when the hazard curves are proportional to, non-proportional to, or even crossing over one another, the proposed MIT can maintain reasonably accurate type I error rates for censored data. It has far greater powers than the modified RERI and PRISM tests (modified for censored data scenarios). To test mechanistic interactions in censored data, we recommend using MIT in light of its desirable statistical properties.

## Introduction

The assessment of interactions is important for epidemiologists and clinicians alike. Epidemiologists are interested in knowing whether the combined effect of two risk factors is greater than the effect expected when considering these same risk factors separately. For example, Jensen et al [1] found that the combination of obesity and heavy smoking creates a risk for acute coronary syndrome which is greater than the risk posed by either of these factors in isolation. On the other hand, clinicians are interested in knowing whether a particular subgroup of patients will benefit more from a new therapeutic agent. For example, Tsao et al [2] found that the presence of an EGFR mutation may increase responsiveness to erlotinib for patients with non-small-cell lung cancer. In both of these examples, the combined effect of two factors indeed exceeds the expectations of one factor alone. However, we might question whether this means that the two factors interact mechanistically to bring about the outcome?

The sufficient cause framework conceptualizes causation as a collection of causal mechanisms, each requiring component causes to operate [3]. If two factors participate in the same causal mechanism, we will say there is synergism between the two factors in the sufficient cause sense, i.e., causal co-action, causal mechanistic interaction, or simply *mechanistic* interaction (a term we adopted in this paper). This should not be confused with the *multiplicative* interaction often assessed by including a cross-product term in a multiplicative model. The (multiplicative) Cox model which assumes proportional hazards is probably the statistical method most commonly used in follow-up data. The use of the Cox model is currently so prevalent that many epidemiologists and clinicians will mistake a significant multiplicative interaction in a Cox model as indicating a genuine mechanistic interaction.

Recently, indices specific to mechanistic interactions have been proposed, including the ‘relative excess risk due to interaction’ (RERI) [4–7] and the ‘peril ratio index of synergy based on multiplicativity’ (PRISM) [8]. RERI is an index based on risk-ratio additivity, and a mechanistic interaction can be declared when RERI is statistically larger than one. PRISM is an index based on peril-ratio multiplicativity, and a mechanistic interaction can be declared when log PRISM is statistically different from zero. PRISM has a less stringent threshold to detect mechanistic interactions as compared to RERI [8]. Both indices assume no loss to follow up and no competing death in a study. But in fact, data censoring (due to loss to follow up, competing death, or simply study closure) is inevitable in any long-term follow-up study.

In this paper, we propose a novel statistical test, the ‘mechanistic interaction test’ (MIT), for censored data. We will examine the statistical properties of the MIT using Monte-Carlo simulations and demonstrate its use based upon real data.

## Sufficient Causes and Mechanistic Interactions

**Fig 1:**
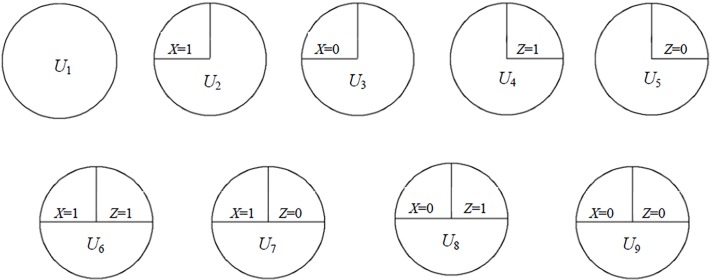
The total 9 classes of sufficient causes for two dichotomous exposures.

In this section, we give an overview of the sufficient cause framework and its relation to mechanistic interactions. In a sufficient component cause model, a sufficient cause contains a combination of component causes. Any sufficient cause with all components completed is sufficient to cause the disease. For two dichotomous exposures (*X* and *Z*), sufficient causes can be classified into a total of nine classes, including one ‘all-unknown’ class (*U*_1_), two *X*-only classes (*U*_2_ and *U*_3_), two *Z*-only classes (*U*_4_ and *U*_5_), and four interaction classes (*U*_6_∼*U*_9_) (see Fig. 1) [8, 9]. We will say there is mechanistic interaction between *X* and *Z*, if at least one of the four interaction classes is present.

In the assessment of mechanistic interactions, RERI has recently received much attention. For two dichotomous exposures, RERI = RR_1,1_ − RR_1,0_ − RR_0,1_ + 1, where RR_*x*,*z*_ is the risk ratio comparing the (cumulative) disease risks (in a certain time interval) between those with exposure profile of (*X* = *x*, *Z* = *z*) and those with (*X* = 0, *Z* = 0), i.e., ${\text{RR}}_{x,z}=\frac{{\text{Risk}}_{x,z}}{{\text{Risk}}_{0,0}}$. A test for RERI > 1 is a specific test for the *U*_6_ (“*X* = 1” × “*Z* = 1”) interaction class [4–7].

Lee [8] pointed out that as follow-up time approaches infinity, RR_*x*,*z*_ will tend toward a value of one for each and every *x*, *z* ∈ {0, 1}, RERI will tend toward zero (perfect risk-ratio additivity), and at this point a test for RERI > 1 will have no hope of achieving significance. Lee [8] therefore proposed an alternative metric of risk: the ‘peril’ which is simply an exponentiated cumulative rate or an inverse of a survival proportion (risk complement). The ‘peril ratio index of synergy based on multiplicativity’ (PRISM) was defined as $\text{PRISM}=\frac{{\text{Peril}}_{\text{1,1}}\times {\text{Peril}}_{\text{0,0}}}{{\text{Peril}}_{\text{1,0}}\times {\text{Peril}}_{\text{0,1}}}=\frac{{\left({\text{1-Risk}}_{1,1}\right)}^{-1}\times {\left({\text{1-Risk}}_{0,0}\right)}^{-1}}{{\left({\text{1-Risk}}_{1,0}\right)}^{-1}\times {\left({\text{1-Risk}}_{0,1}\right)}^{-1}}.$ Under the no-redundancy assumption [9], Lee [8] showed that a test for log PRISM ≠ 0 is a global test for mechanistic interaction (at least one of *U*_6_∼ *U*_9_ is present).

## Mechanistic Interaction Test

In a follow-up study, we assume that the exposure status is time-invariant. Note that here we allow the study to have censored observations, provided that the censoring mechanism is independent of the event process. Assuming that there is no confounding, selection bias, or measurement error in the study, the association between the two exposures and the disease should reflect the genuine causal effect of the exposures. Let the hazard rate at follow-up time *t* for people with exposure profile of *X* = *x* and *Z* = *z* be denoted by *h*_*x*,*z*_(*t*). Define the ‘interaction contrast’ (IC) as an index of departure from hazard-rate additivity, that is, IC(*t*) = *h*_1,1_(*t*) − *h*_1,0_(*t*) − *h*_0,1_(*t*) + *h*_0,0_(*t*) at follow-up time *t*. As shown in Lee [8, 9], IC(*t*) = 0 for every *t* under the null hypothesis of no *mechanistic* interaction. [By contrast, *multiplicative* interaction is assessed by departure from hazard-rate multiplicativity (such as in Cox regression), that is, $\phi (t)=\frac{h_{1,1}(t)}{h_{0,1}(t)}/\frac{h_{1,0}(t)}{h_{0,0}(t)}$ at follow-up time *t*. Under the null hypothesis of no multiplicative interaction, *ϕ*(*t*) = 1 for every *t* [10]. For more elaboration on the difference between mechanistic and multiplicative interactions, see S1 Appendix.]

Assume that a follow-up study yields a total of *J* (*j* = 1,2,…*J*) ‘risk sets’. (A risk set is the set of subjects contracting a disease at a particular point in time, together with all those subjects at risk for the disease at that time.) Define ${\text{IC}}_{j}=\frac{d_{11j}}{n_{11j}}-\frac{d_{10j}}{n_{10j}}-\frac{d_{01j}}{n_{01j}}+\frac{d_{00j}}{n_{00j}}$ for *j* = 1,2,…*J*, where (*n*_x,z,j_) is the number of diseased subjects (subjects at risk) with an exposure profile of *X* = *x* and *Z* = *z* at the *j* th risk set. Under the null hypothesis, we therefore have E(IC_*j*_) = 0 for *j* = 1,2,…*J*. The proposed MIT is defined as

$$\text{MIT}=\frac{{\left(\sum_{j=1}^{J}w_{j}\times {\text{IC}}_{j}\right)}^{2}}{\sum_{j=1}^{J}{\left(w_{j}\times {\text{IC}}_{j}\right)}^{2}},$$

where *w*_*j*_ is the weight for the *j*th risk set (to be discussed shortly). Asymptotically for large *J* (e.g., *J* > 30, that is, large number of risk sets), MIT is distributed as a chi-square distribution with one degree of freedom under the null hypothesis of no mechanistic interaction. Like the PRISM test [8], the proposed MIT is a global test; a significant test result implies the presence of at least one of the four interaction classes.

A sensible choice of the weight is the harmonic sum of the sample sizes of the four exposure profiles, $w_{j}={\left(\frac{1}{n_{11j}}+\frac{1}{n_{10j}}+\frac{1}{n_{01j}}+\frac{1}{n_{00j}}\right)}^{-1}.$ When hazard rates are approximately the same for each exposure profile at each follow-up time, the weight is inversely proportional to the variance of IC_*j*_. Such a weighting system thus makes optimal use of the information contained in different risk sets.

## Simulation Studies

**Fig 2:**
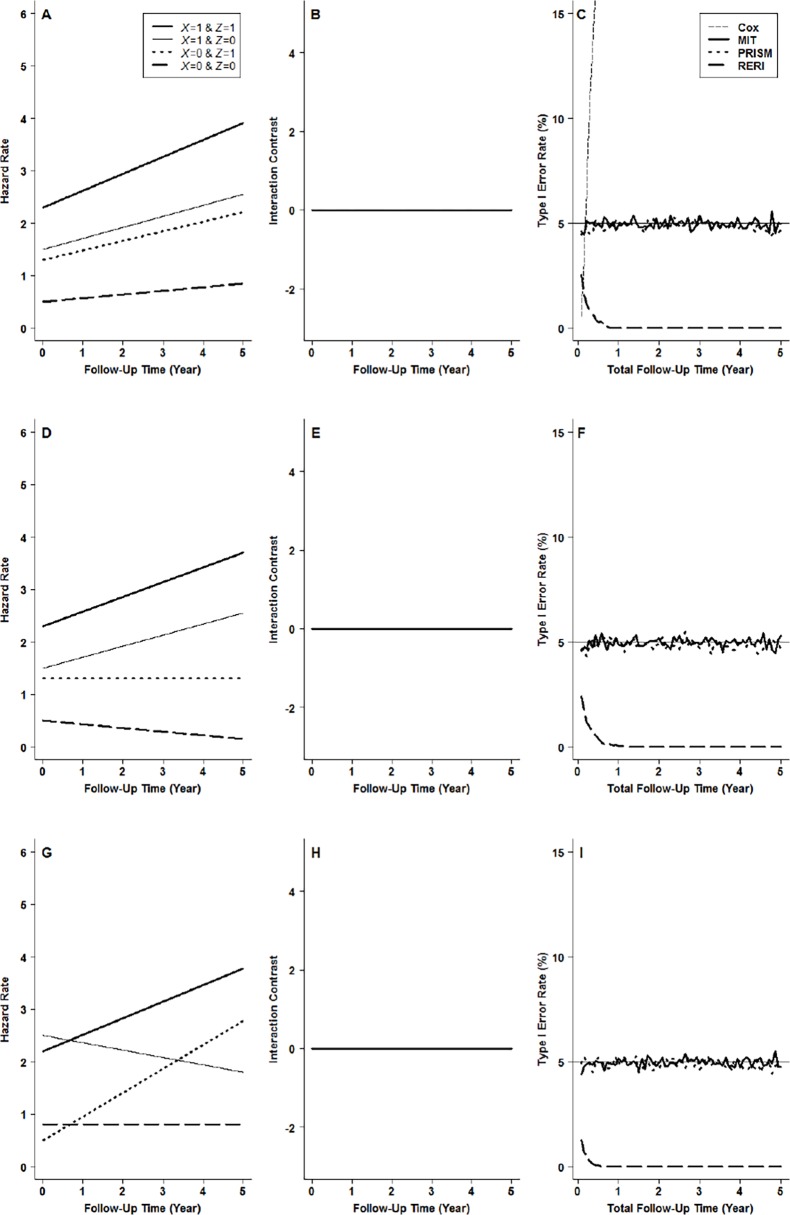
Type I error rates under the null hypothesis of no mechanistic interaction: interaction contrast (B) and type I error rates (C) for proportional hazards (A), interaction contrast (E) and type I error rates (F) for non-proportional hazards (D), and interaction contrast (H) and type I error rates (I) for crossover hazards (G). Bold solid lines in A, D and G are hazard rates for people with exposure profile of *X* = 1 and *Z* = 1, thin solid lines, those for *X* = 1 and *Z* = 0, bold dotted lines, those for *X* = 0 and *Z* = 1, and bold broken lines, those for *X* = 0 and *Z* = 0. Bold solid lines in C, F and I are for MIT (mechanistic interaction test), bold dotted lines, for PRISM (peril ratio index of synergy based on multiplicativity), bold broken lines, for RERI (relative excess risk due to interaction), and the horizontal thin solid lines, the nominal *α* level of 0.05. The thin broken line in C is for the interaction test in the Cox model.

**Fig 3:**
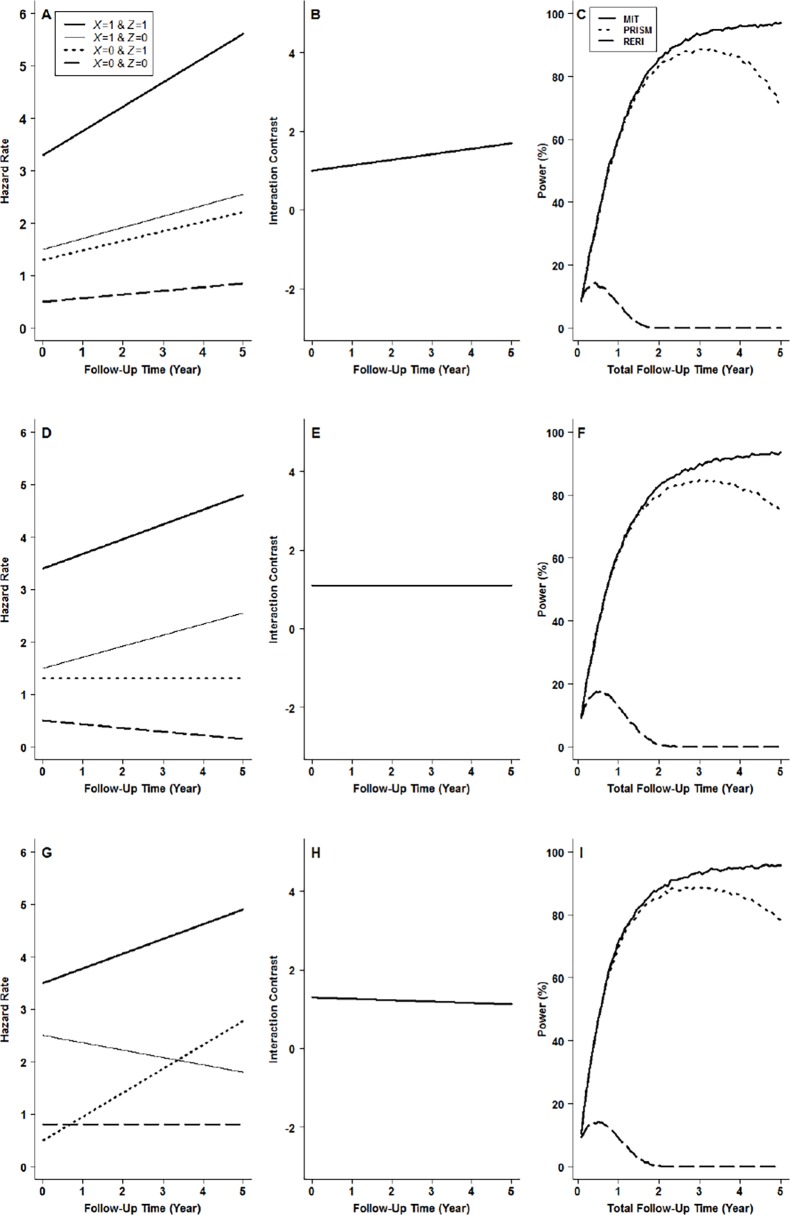
Empirical powers under the alternative hypothesis of mechanistic interactions: interaction contrast (B) and powers (C) for proportional hazards (A), interaction contrast (E) and powers (F) for non-proportional hazards (D), and interaction contrast (H) and powers (I) for crossover hazards (G). Bold solid lines in A, D and G are hazard rates for people with exposure profile of *X* = 1 and *Z* = 1, thin solid lines, those for *X* = 1 and *Z* = 0, bold dotted lines, those for *X* = 0 and *Z* = 1, and bold broken lines, those for *X* = 0 and *Z* = 0. Bold solid lines in C, F and I are for MIT (mechanistic interaction test), bold dotted lines, for PRISM (peril ratio index of synergy based on multiplicativity), and bold broken lines, for RERI (relative excess risk due to interaction).

Monte-Carlo simulation was conducted to evaluate the performance of MIT. For simplicity, the hazard rate for each and every exposure profile is assumed to be a linear function of *t* (detailed in S2 Appendix). (S3 Appendix presents the simulation results for more complex hazard functions. The results are essentially the same as those presented in this paper.) To obtain a complete picture of the performance of MIT, we deliberately contrived scenarios where the hazard curves for the four exposure profiles were proportional to one another (Panel A in Figs. 2 and 3), where they were non-proportional (Panel D in Figs. 2 and 3), and where non-proportionality was so extreme that some of the hazard curves actually crossed others (Panel G in Figs. 2 and 3). We considered all those scenarios where the hazard curves satisfied IC(*t*) = 0 for every *t* (null hypothesis of no mechanistic interaction, shown in Panels B, E and H in Fig. 2) as well as when they did not (alternative hypothesis, shown in Panels B, E and H in Fig. 3). We assumed 250 subjects for each exposure profile at the beginning of a five-year follow-up study. The censoring process was assumed to follow a simple exponential distribution with a hazard rate of 0.2. A total of 10000 simulations were performed for each scenario. In each round of the simulation, MITs with different total follow-up times were calculated. The level of significance was set at *α* = 0.05.

For a comparison, we also performed the interaction test in a Cox model (only for the proportionality scenarios), the RERI and the PRISM tests (modified for censored data scenarios in this paper, detailed in S4 Appendix). For the Cox model, we tested the cross-product term of the two exposures. For RERI, we tested (one-sided) whether the index was statistically larger than one (a specific test for *U*_6_), and for PRISM, we tested (two-sided) whether log PRISM was statistically different from zero (a global test for *U*_6_∼*U*_9_).

Fig. 2 shows the type I error rates for the scenarios of proportionality (Panel C), non-proportionality (Panel F), and crossover (Panel I), respectively. It can be seen that for the MIT and PRISM, the type I error rates with different total follow-up time are very close to the nominal *α* level for all scenarios. [MIT and PRISM can still maintain accurate type I error rates even when subjects with different exposure profiles have different censoring rates (see S5 Appendix).] By contrast, RERI is a very conservative test with unduly small type I errors. As for the interaction test in the Cox model, its type I error rates are severely inflated even under the proportionality assumption (Fig. 2C), and we will thus not consider it further in the following power analysis.

Fig. 3 (Panels C, F, and I) shows the simulation results for the powers. In all scenarios, the power of the proposed MIT increases as the total follow-up time increases and is higher than those of the remaining two competitors (PRISM and RERI). The power of the PRISM test initially matches that of the MIT and it also increases as total follow-up time increases. Yet, as the total follow-up time passes a certain threshold (3 years when around 80% of the subjects become diseased), the power decreases instead. As for the RERI test, it has minimal power to detect mechanistic interactions; even so this is limited to only a very early phase of the follow-up (< 1.5 years).

MIT also reveals the desirable statistical properties in further simulation with smaller (S6 Appendix), and unbalanced (i.e., unequal numbers of subjects in different exposure profiles, S7 Appendix), sample sizes.

## An Example

Klein and Moeschberger described a follow-up study of allogeneic marrow transplantation for patients with acute leukemia (the Appendix D in reference 10). A total of 137 patients from March 1, 1984 were followed up. We restricted our analysis in the five-year period afterward. During this period, 42 patients relapsed (two patients relapsed at the same time). A total of 41 risk sets can be defined (see Table 1). Several potential risk factors were measured at the time of transplantation, including recipient cytomegalovirus (CMV) immune status (coded as 1 if CMV positive and 0 if otherwise) and use of a graft-versus-host prophylaxis regimen of methotrexate (MTX) (coded as 1 if MTX was used, and 0 if it was not).

**Table 1:** The total 41 risk sets for the example data (taken from Appendix D in reference 10).

Ordered Risk Set	Failure Time	Number of Subjects At Risk				Risk Factor Status for the Relapsed Patients
(*j*)	(Days)	*n*_11*j*_	*n*_10*j*_	*n*_01*j*_	*n*_00*j*_	(CMV^a^, MTX^b^)
1	32	26	40	12	55	(0,0)
2	47	25	40	12	54	(0,0) and (0,0)
3	48	25	40	12	52	(0,0)
4	55	24	39	12	51	(1,1)
5	64	23	39	11	51	(1,1)
6	74	22	39	11	51	(1,1)
7	76	20	39	11	51	(1,1)
8	84	19	39	10	49	(1,0)
9	93	18	38	10	49	(1,0)
10	100	18	37	10	49	(1,0)
11	104	18	36	10	49	(0,0)
12	109	17	35	10	47	(1,1)
13	110	16	35	10	47	(1,1)
14	113	15	35	10	47	(1,1)
15	115	14	35	10	47	(1,0)
16	120	14	34	10	47	(1,0)
17	122	14	33	10	47	(1,1)
18	129	13	33	10	46	(0,1)
19	157	13	33	9	46	(1,0)
20	192	12	32	9	42	(1,1)
21	211	11	32	9	41	(0,0)
22	219	11	32	9	40	(1,1)
23	230	10	32	9	39	(0,0)
24	242	10	32	9	38	(0,0)
25	248	10	32	9	37	(0,0)
26	268	10	32	9	36	(1,0)
27	272	10	31	9	36	(1,0)
28	273	10	30	9	36	(1,1)
29	381	8	28	8	35	(0,0)
30	383	8	28	8	34	(0,0)
31	390	8	28	8	33	(1,0)
32	421	8	27	8	30	(0,0)
33	422	8	27	8	29	(0,0)
34	456	8	27	8	28	(1,0)
35	467	8	26	7	28	(1,0)
36	486	8	24	7	28	(0,0)
37	606	8	24	6	25	(1,1)
38	609	7	24	6	25	(1,0)
39	625	7	23	6	25	(1,0)
40	662	7	22	6	24	(0,0)
41	748	7	20	6	23	(0,0)

^a^ Cytomegalovirus immune status: 1 if CMV positive, and 0 if otherwise.

^b^ Methotrexate usage: 1 if MTX being used, and 0 if otherwise.

We used the Kaplan-Meier method to estimate the 5-year cumulative relapse-free proportions for people with different exposure profiles (of CMV status and MTX treatment). The cumulative relapse risks were then 1 − 0.4172 = 0.5828 for the (CMV = 1, MTX = 1) subgroup, 1 − 0.6476 = 0.3524 for the (CMV = 1, MTX = 0) subgroup, 1 − 0.9000 = 0.1000 for the (CMV = 0, MTX = 1) subgroup, and 1 − 0.6363 = 0.3637 for the (CMV = 0, MTX = 0) subgroup, respectively. It is of interest to note that CMV status appears to modify the effect of MTX. For CMV-negative patients, MTX treatment reduces the risk of leukemic relapse from 0.3633 to 0.1000, whereas for CMV-positive patients, the same treatment increases the risk from 0.3524 to 0.5828. But is there a mechanistic interaction between CMV status and MTX treatment?

Using the method proposed in this paper, we calculated the MIT statistic of this example (based on the risk-set data presented in Table 1) to be 6.2323 with a *P* value of 0.0125. We therefore have evidences for a mechanistic interaction between CMV status and MTX treatment. For this example, the PRISM index (based on Kaplan-Meier estimates) is $\left(\frac{1}{0.4172}\times \frac{1}{0.6363}\right)/\left(\frac{1}{0.6476}\times \frac{1}{0.9000}\right)=2.1957$ with a slightly larger *P* value of 0.0189. The RERI index (based on Kaplan-Meier estimates) is (0.5828 − 0.3524 − 0.1000 + 0.3637)/0.3637 = 1.3585 with a much larger and non-significant *P* value of 0.2173. Again we see that the RERI test is underpowered. The interaction test using the Cox regression results in a marginally non-significant *P* value of 0.0521 for this example. But we caution again that this is a test for multiplicative interaction and not a test for mechanistic interaction.

## Discussion

Epidemiologists had long been pondering over the possibility of testing mechanistic interactions using only the observational data at hand. Recent developments in causal-inference methodologies had for the first time proved that this was indeed possible for cohort and case-control data [4–8]. However, testing mechanistic interactions in censored data is somewhat complicated. A stratified log-rank test can be used to test the equality of two survival distributions (of the exposed and the unexposed) in each and every stratum delineated by another variable. This, however, is still a test to determine the main effect of the exposure but is not a test to measure interactions. A modification of the Cox model (such that hazard ratios are a linear function of covariates) can test for mechanistic interactions [9] but requires the proportional hazards assumption. By contrast, the proposed MIT is extremely robust. It can test mechanistic interactions when the hazard curves are proportional to, non-proportional to, or even crossing over, one another.

As mentioned previously, a test for RERI > 1 is a specific test for the *U*_6_ interaction class [4–7]. In our simulation studies, RERI test is severely underpowered. Under a strong assumption, the ‘monotonicity assumption’ [11–14], *U*_6_ is the only interaction class to consider and the threshold of the RERI test becomes less stringent (RERI > 0 vs. RERI > 1) [4–7]. Even with this additional assumption, however, the power of the test for RERI > 0 still decreases very quickly as total follow-up time increases (S8 Appendix). The limited power of the RERI test (whether for RERI > 0 or RERI > 1) is understandable, because it assesses departure from risk-ratio additivity at one and only one point in time: the time of study closure. By contrast, the (weighted) sum operation in MIT integrates information at various time points during the follow-up (from the different risk sets, each one gauging a departure from hazard-rate additivity at a specific point in time). Comparatively it therefore has much greater power to detect mechanistic interactions.

In this study, we found that the power of the PRISM test initially matches that of MIT, but beyond a certain point (when ∼80% of the subjects are diseased, see also S7 in reference 8), the power does not increase but instead can actually decrease with more follow-up time. This is rather undesirable; with more efforts put into longer-term follow-up, a researcher naturally will expect the study to generate more power—but not less—if at all! We show in S9 Appendix that the PRISM test is akin to a hazard-rate additivity test with equal weight attached to the risk sets (cf. the adaptive weight in MIT according to the sample size of a set). As follow-up progresses, the size of a risk set will gradually decrease to a point where it contains too few subjects to provide any reliable information regarding hazard-rate additivity. Indiscriminately assigning large weightings to a limited number of subjects will only overwhelm the true interaction signals that may be present in the earlier sets.

To test for mechanistic interactions between two binary exposures in long-term follow-up studies, we recommend using MIT in light of its desirable statistical properties. Further studies are warranted to expand the methodologies to include categorical or ordinal variables with three or more levels. A stratified MIT also awaits development which should prove useful to adjust for possible confounders (stratification according to the levels of confounders). Finally, further work is needed to cast the present method in a general regression framework.

## Supporting Information

S1 Appendix Comparison between mechanistic and multiplicative interactions. (DOC)

S2 Appendix The linear function of the hazard rate for each and every exposure profile. (DOC)

S3 Appendix Simulation results for more complex hazard functions. (DOC)

S4 Appendix Modified RERI and the PRISM tests for censored data scenarios. (DOC)

S5 Appendix Simulation results when subjects with different exposure profiles have different censoring rates. (DOC)

S6 Appendix Simulation results for smaller sample sizes. (DOC)

S7 Appendix Simulation results for unbalanced sample sizes (across different exposure profiles). (DOC)

S8 Appendix Simulation results for the RERI test under the assumption of monotonicity. (DOC)

S9 Appendix The PRISM test: a hazard-rate additivity test with equal weight. (DOC)
