# Supplementary material for: Testing for Mechanistic Interactions in Long-Term Follow-Up Studies
Source: PLoS One. 2015 Mar 26;10(3):e0121638. doi: 10.1371/journal.pone.0121638 (PMC4374952; doi:10.1371/journal.pone.0121638)

**S1 Appendix.**

I. An example with multiplicative interaction [ for some $t$] but without mechanistic interaction [$\text{IC}(t)=0$ for every $t$]:


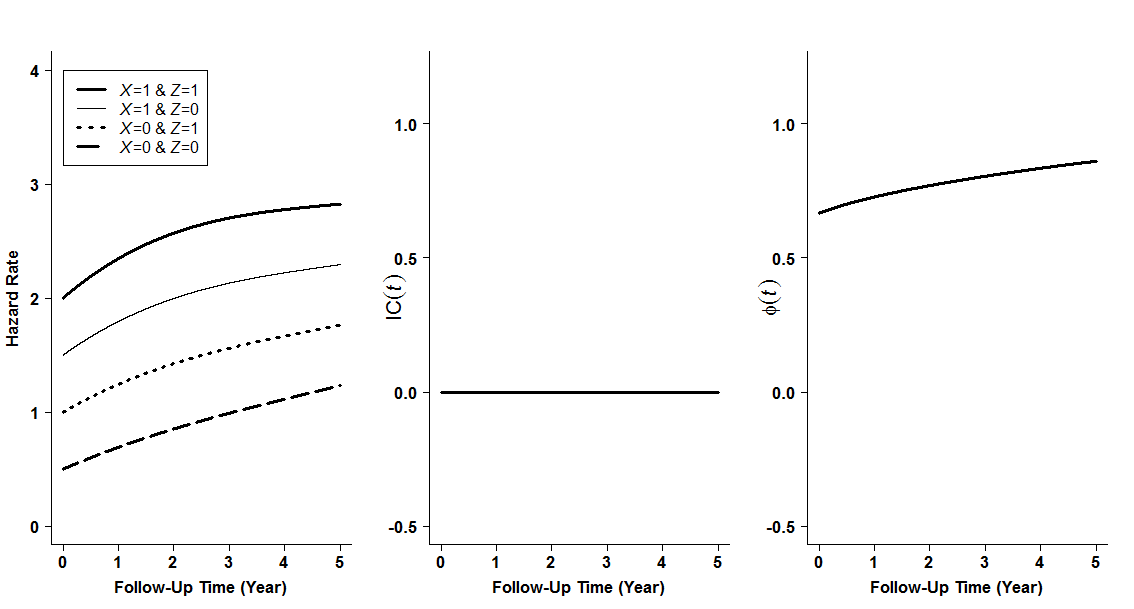


II. An example with mechanistic interaction [$\text{IC}(t)\neq0$ for some $t$] but without multiplicative interaction [ for every $t$]:


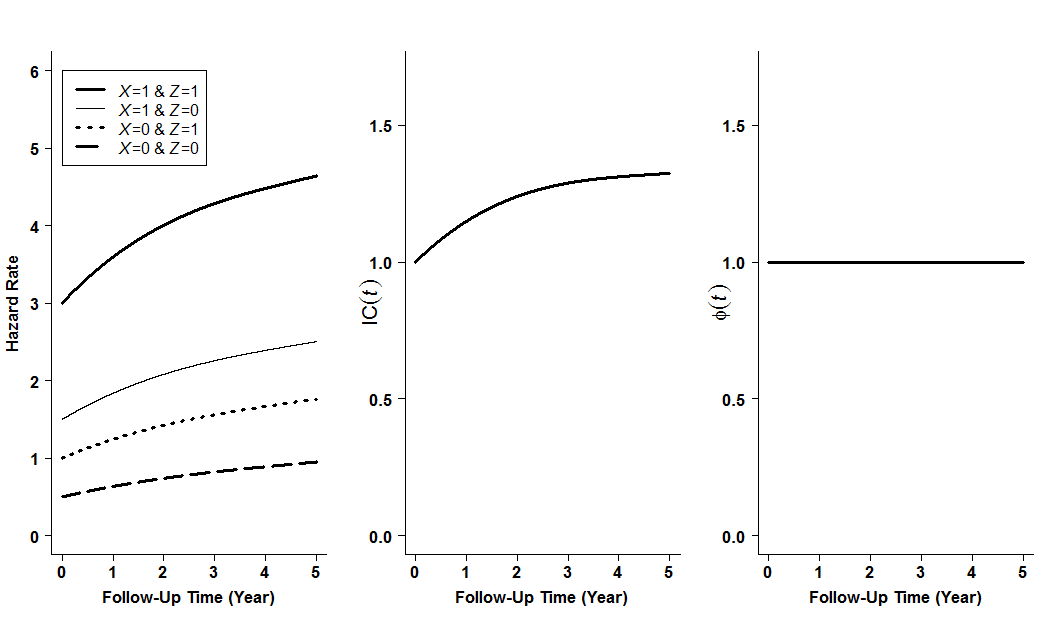

Supplement: S1 Appendix — (DOC) [file pone.0121638.s001.doc]
