# Supplementary material for: Testing for Mechanistic Interactions in Long-Term Follow-Up Studies
Source: PLoS One. 2015 Mar 26;10(3):e0121638. doi: 10.1371/journal.pone.0121638 (PMC4374952; doi:10.1371/journal.pone.0121638)
Supplement: S2 Appendix — (DOC) [file pone.0121638.s002.doc]

**S2 Appendix.**

In simulation studies, the hazard rate for each and every exposure profile is assumed to be a linear function of $t$. Figure 2 is for the null hypothesis, that is, $\text{IC}(t)=h_{1,1}(t)-h_{1,0}(t)-h_{0,1}(t)+h_{0,0}(t)=0$ for every $t$. For proportional hazards (Panel A), we let $h_{1,1}(t)=0.322\times t+2.3$, $h_{1,0}(t)=0.21\times t+1.5$, $h_{0,1}(t)=0.182\times t+1.3$, and $h_{0,0}(t)=0.07\times t+0.5$. For non-proportional hazards (Panel D), we let $h_{1,1}(t)=0.28\times t+2.3$, $h_{1,0}(t)=0.21\times t+1.5$, $h_{0,1}(t)=1.3$, and $h_{0,0}(t)=-0.07\times t+0.5$. For crossover hazards (Panel G), we let $h_{1,1}(t)=0.315\times t+2.2$, $h_{1,0}(t)=-0.14\times t+2.5$, $h_{0,1}(t)=0.455\times t+0.5$, and $h_{0,0}(t)=0.8$. Figure 3 is for the alternative hypothesis, that is, $\text{IC}(t)=h_{1,1}(t)-h_{1,0}(t)-h_{0,1}(t)+h_{0,0}(t)\neq0$ for some $t$. Here we replaced $h_{1,1}(t)$ of the above three scenarios under the null hypothesis with $h_{1,1}(t)=0.462\times t+3.3$ (Panel A), $h_{1,1}(t)=0.28\times t+3.4$ (Panel D), and $h_{1,1}(t)=0.28\times t+3.5$ (Panel G), respectively.
