# Supplementary material for: Testing for Mechanistic Interactions in Long-Term Follow-Up Studies
Source: PLoS One. 2015 Mar 26;10(3):e0121638. doi: 10.1371/journal.pone.0121638 (PMC4374952; doi:10.1371/journal.pone.0121638)

**S3 Appendix.**

Here we present the simulation results for more complex hazard functions. The hazard rate for each and every exposure profile is assumed to be a piecewise function (Part 1), a quadratic function (Part 2), and an exponential function (Part 3) of $t$.

Under the null hypothesis of no mechanistic interaction, the following figures show interaction contrast (B) and type I error rates (C) for proportional hazards (A), interaction contrast (E) and type I error rates (F) for non-proportional hazards (D), and interaction contrast (H) and type I error rates (I) for crossover hazards (G).

Under the alternative hypothesis of mechanistic interaction, the following figures show interaction contrast (B) and powers (C) for proportional hazards (A), interaction contrast (E) and powers (F) for non-proportional hazards (D), and interaction contrast (H) and powers (I) for crossover hazards (G).

Part 1. Piecewise function

In simulation studies, the hazard rate for each and every exposure profile is assumed to be a piecewise function of $t$. In the following functions, *I*(statement), an indicator function, has a value of 1, if the statement is true, and a value of 0, if otherwise. Under the null hypothesis, for proportional hazards (Panel A), we let $h_{1,1}(t)=2\times I\left(0\leq t<2\right)+2.5\times I\left(2\leq t\leq5\right)$, $h_{1,0}(t)=1.5\times I\left(0\leq t<2\right)+1.875\times I\left(2\leq t\leq5\right)$, $h_{0,1}(t)=1\times I\left(0\leq t<2\right)+1.25\times I\left(2\leq t\leq5\right)$, and $h_{0,0}(t)=0.5\times I\left(0\leq t<2\right)+0.625\times I\left(2\leq t\leq5\right)$.

For non-proportional hazards (Panel D), we let $h_{1,1}(t)=2\times I\left(0\leq t<1\right)+2.2\times I\left(1\leq t<2\right)+2.4\times I\left(2\leq t\leq5\right)$, $h_{1,0}(t)=1.5\times I\left(0\leq t<3\right)+1.8\times I\left(3\leq t\leq5\right)$, $h_{0,1}(t)=1\times I\left(0\leq t<2\right)+1.2\times I\left(2\leq t<3\right)+0.9\times I\left(3\leq t\leq5\right)$, and $h_{0,0}(t)=0.5\times I\left(0\leq t<1\right)+0.3\times I\left(1\leq t\leq5\right)$. For crossover hazards (Panel G), we let $h_{1,1}(t)=2\times I\left(0\leq t<1\right)+2.5\times I\left(1\leq t<2\right)+2\times I\left(2\leq t\leq5\right)$, $h_{1,0}(t)=1.5\times I\left(0\leq t<2\right)+1\times I\left(2\leq t\leq5\right)$, $h_{0,1}(t)=1\times I\left(0\leq t<1\right)+1.75\times I\left(1\leq t<3\right)+2.25\times I\left(3\leq t\leq5\right)$, and $h_{0,0}(t)=0.5\times I\left(0\leq t<1\right)+0.75\times I\left(1\leq t<3\right)+1.25\times I\left(3\leq t\leq5\right)$. Figure 3 is for the alternative hypothesis, that is, $\text{IC}(t)=h_{1,1}(t)-h_{1,0}(t)-h_{0,1}(t)+h_{0,0}(t)\neq0$ for some $t$. Here we replaced $h_{1,1}(t)$ of the above three scenarios under the null hypothesis with $h_{1,1}(t)=2.8\times I\left(0\leq t<2\right)+3.5\times I\left(2\leq t\leq5\right)$ (Panel A), $h_{1,1}(t)=2.5\times I\left(0\leq t<1\right)+3\times I\left(1\leq t<2\right)+3.5\times I\left(2\leq t\leq5\right)$ (Panel D), and $h_{1,1}(t)=2.8\times I\left(0\leq t<1\right)+3\times I\left(1\leq t<1.5\right)+3.2\times I\left(1.5\leq t\leq5\right)$ (Panel G), respectively. The simulation results are shown below:

1. Under the null hypothesis of no mechanistic interaction


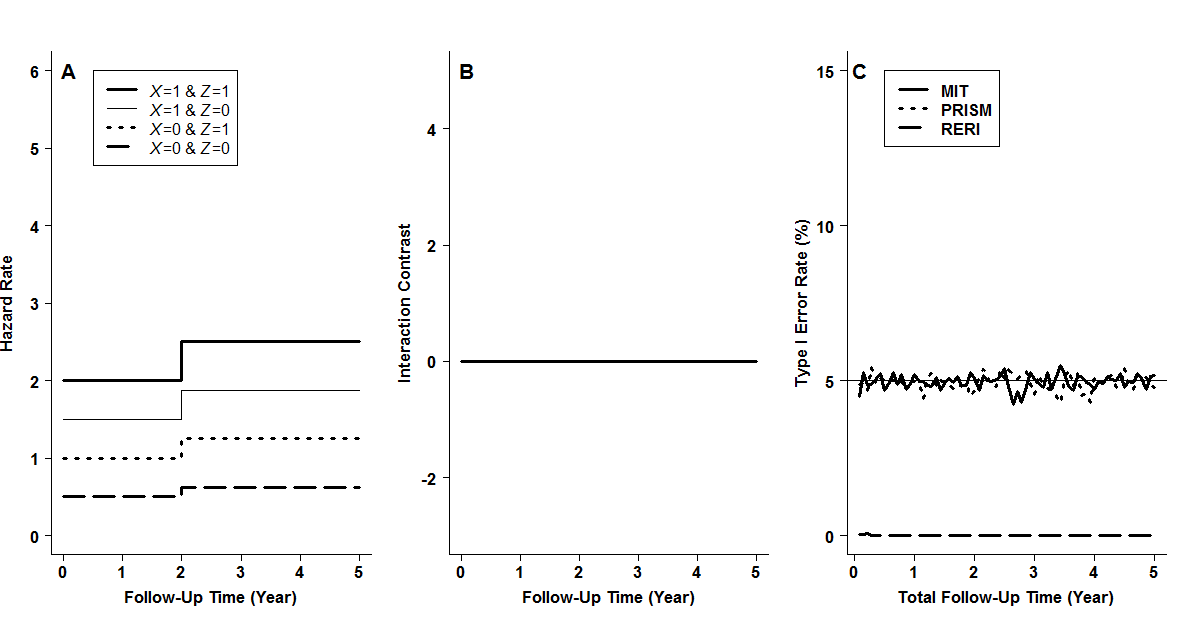


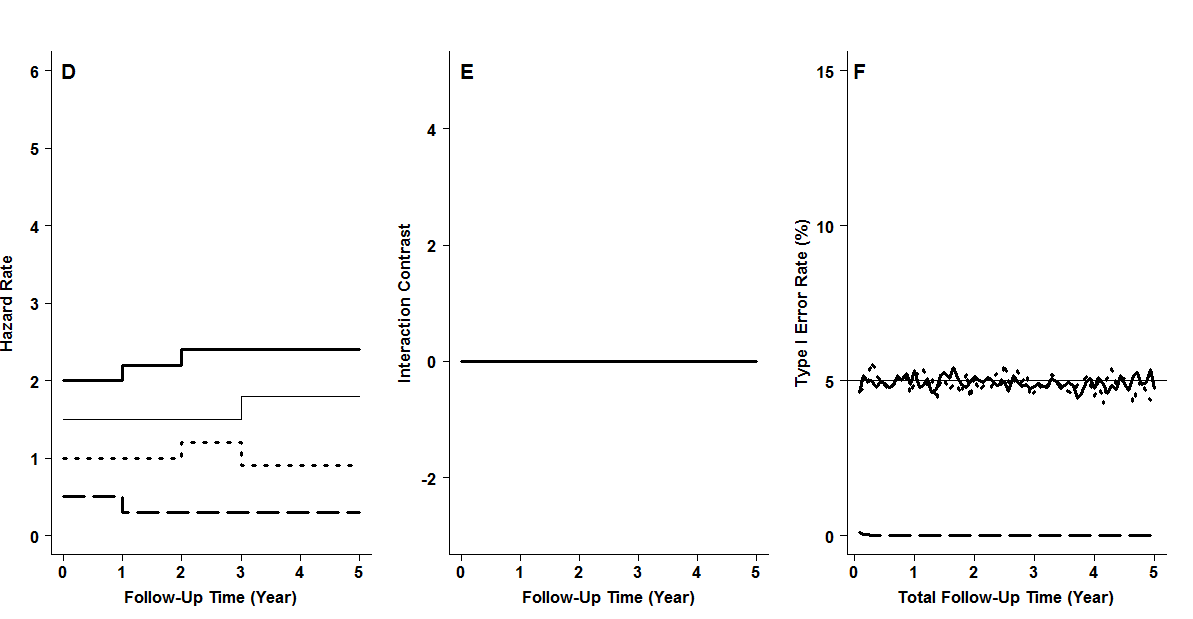


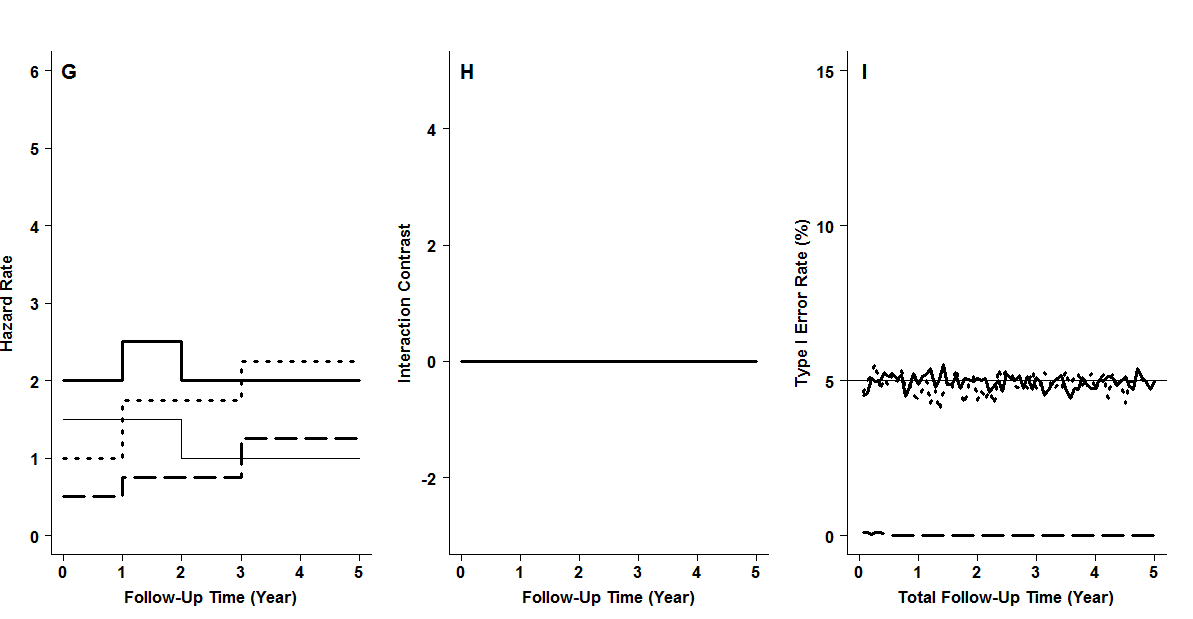


1. Under the alternative hypothesis of mechanistic interaction


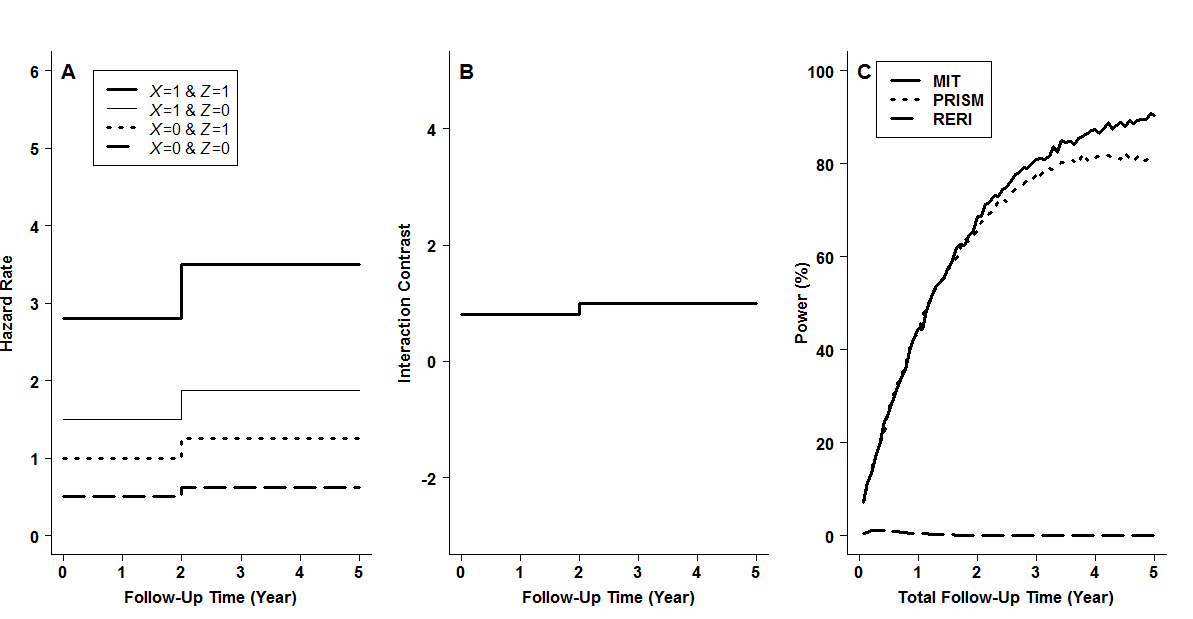


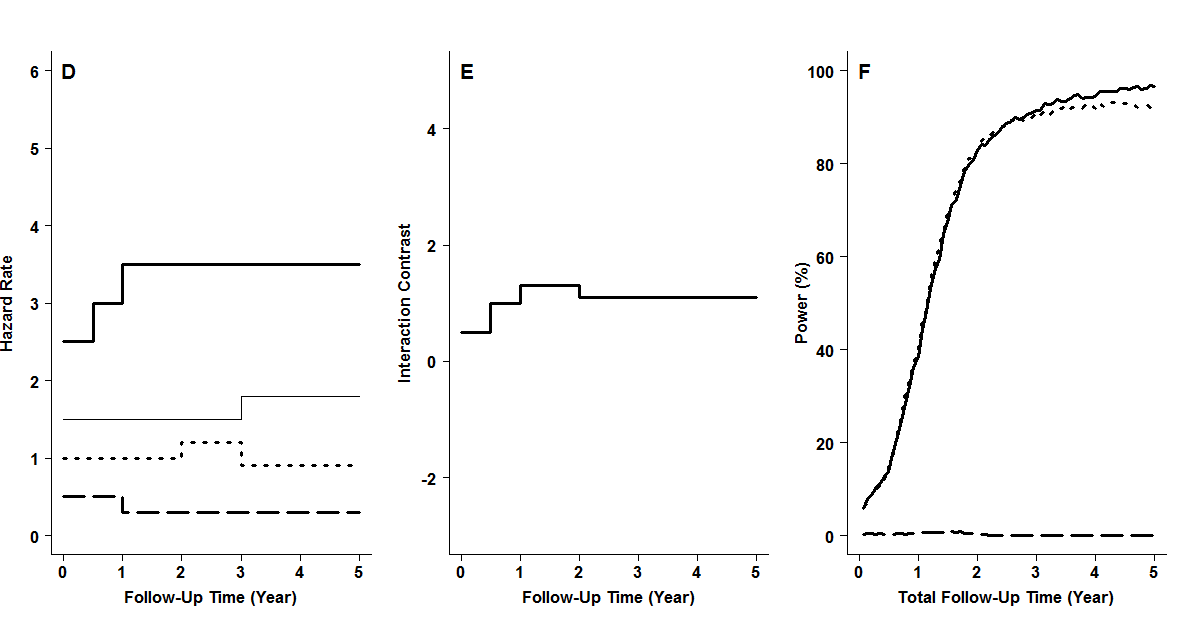


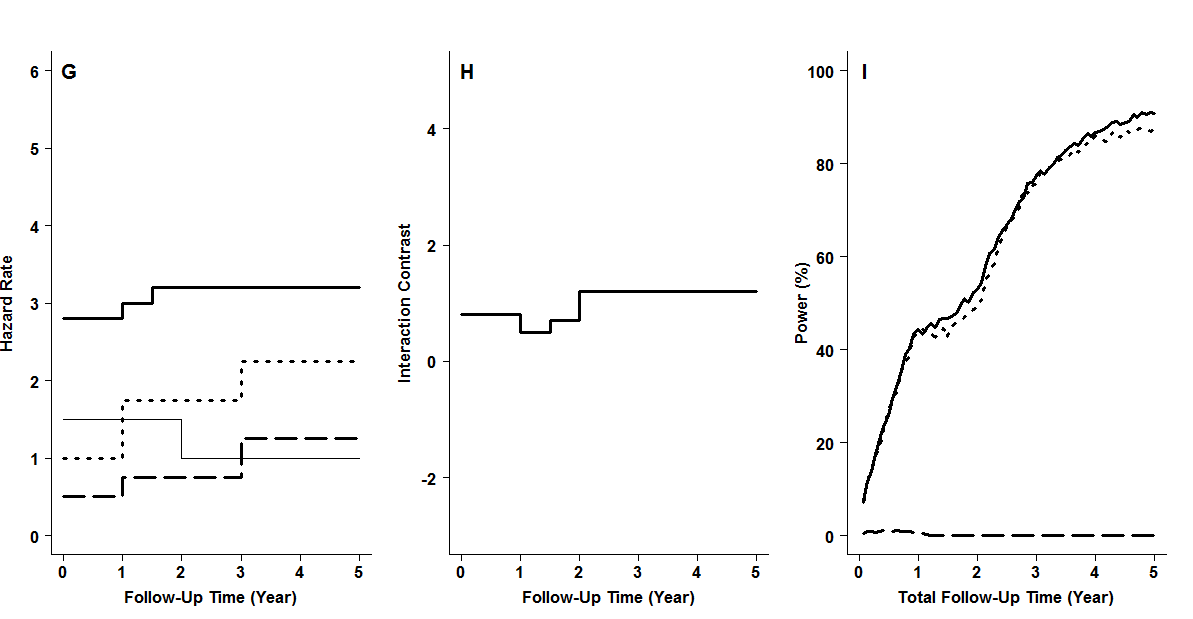


Part 2. Quadratic function

In simulation studies, the hazard rate for each and every exposure profile is assumed to be a quadratic function of $t$. Under the null hypothesis, for proportional hazards (Panel A), we let $h_{1,1}(t)=0.0392\times t^{2}+0.28\times t+2$, $h_{1,0}(t)=0.0294\times t^{2}+0.21\times t+1.5$, $h_{0,1}(t)=0.0196\times t^{2}+0.14\times t+1$, and $h_{0,0}(t)=0.0098\times t^{2}+0.07\times t+0.5$. For non-proportional hazards (Panel D), we let $h_{1,1}(t)=0.0294\times t^{2}+0.28\times t+2.2$, $h_{1,0}(t)=0.0392\times t^{2}+0.21\times t+1.5$, $h_{0,1}(t)=0.14\times t+1.2$, and $h_{0,0}(t)=0.0098\times t^{2}+0.07\times t+0.5$. For crossover hazards (Panel G), we let $h_{1,1}(t)=0.0392\times t^{2}+0.21\times t+1.9$, $h_{1,0}(t)=0.0294\times t^{2}+2.2$, $h_{0,1}(t)=0.0196\times t^{2}+0.28\times t+0.8$, and $h_{0,0}(t)=0.0098\times t^{2}+0.07\times t+1.1$. Figure 3 is for the alternative hypothesis, that is, $\text{IC}(t)=h_{1,1}(t)-h_{1,0}(t)-h_{0,1}(t)+h_{0,0}(t)\neq0$ for some $t$. Here we replaced $h_{1,1}(t)$ of the above three scenarios under the null hypothesis with $h_{1,1}(t)=0.05488\times t^{2}+0.392\times t+2.8$ (Panel A), $h_{1,1}(t)=0.049\times t^{2}+0.392\times t+3$ (Panel D), and $h_{1,1}(t)=0.0588\times t^{2}+0.42\times t+2.8$ (Panel G), respectively. The simulation results are shown below:

1. Under the null hypothesis of no mechanistic interaction


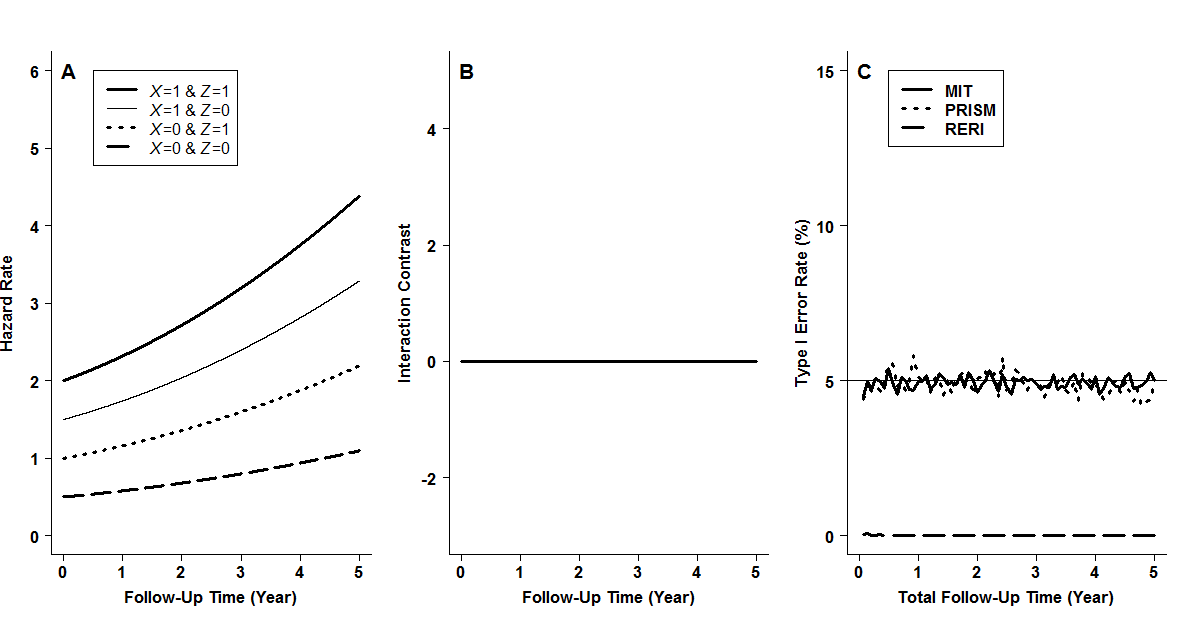


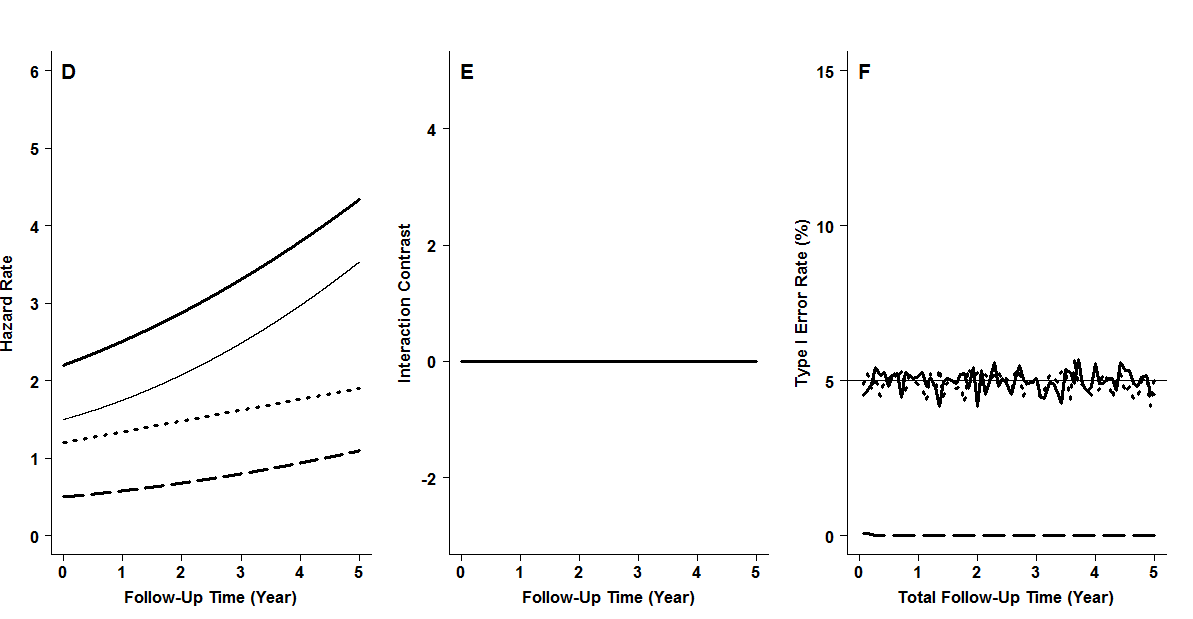


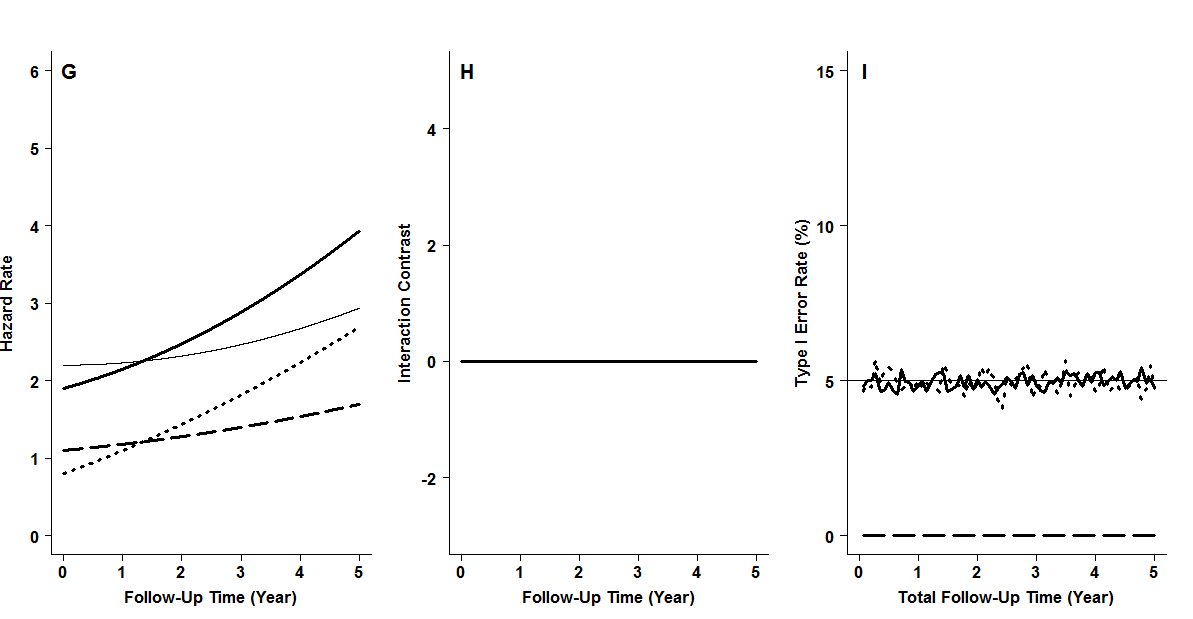


1. Under the alternative hypothesis of mechanistic interaction


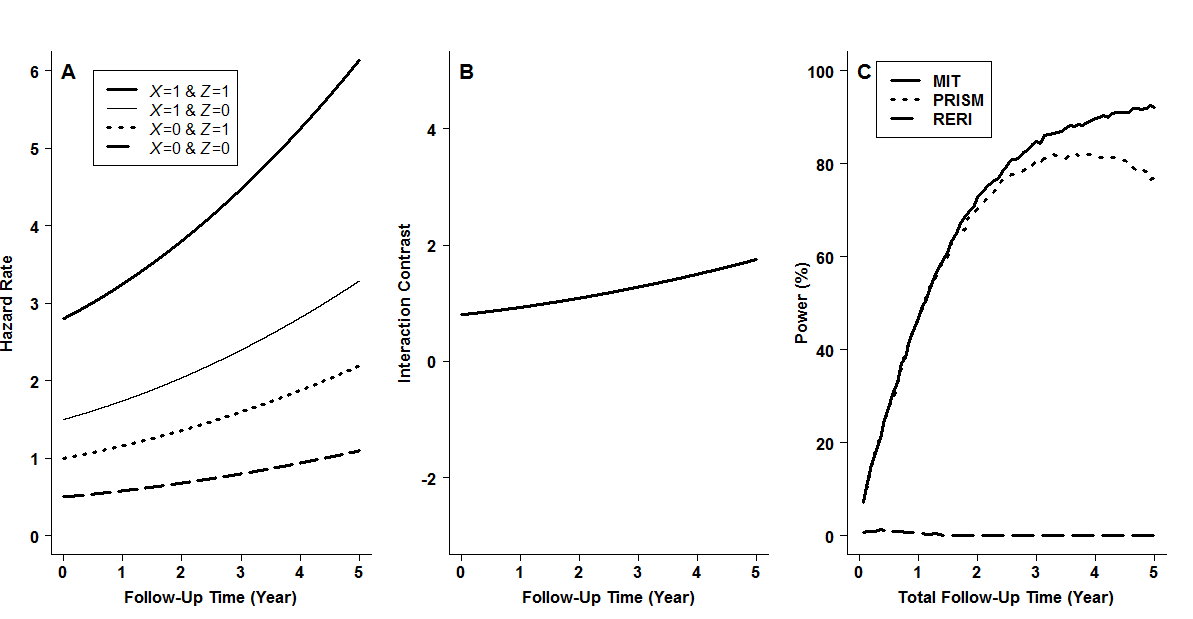


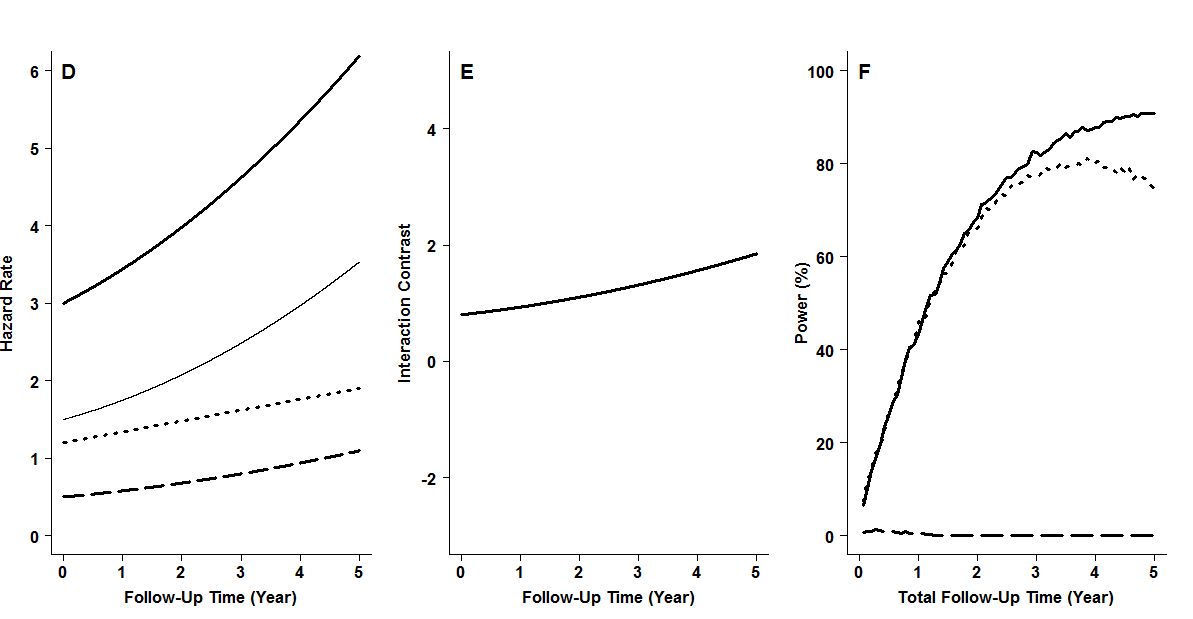


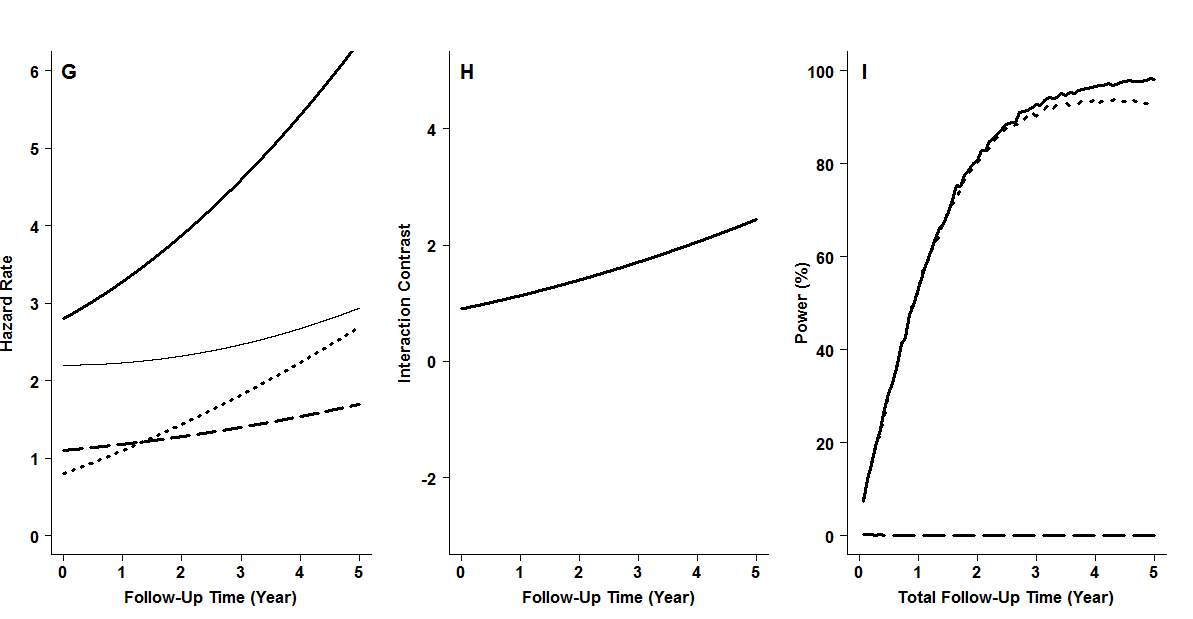


Part 3. Exponential function

In simulation studies, the hazard rate for each and every exposure profile is assumed to be an exponential function of $t$. Under the null hypothesis, for proportional hazards (Panel A), we let $h_{1,1}(t)=2\times\exp\left(0.14\times t\right)$, $h_{1,0}(t)=1.5\times\exp\left(0.14\times t\right)$, $h_{0,1}(t)=\exp\left(0.14\times t\right)$, and $h_{0,0}(t)=0.5\times\exp\left(0.14\times t\right)$. For non-proportional hazards (Panel D), we let $h_{1,1}(t)=\exp\left(0.14\times t\right)+1$, $h_{1,0}(t)=\exp\left(0.14\times t\right)+0.5$, $h_{0,1}(t)=\exp\left(0.14\times t\right)$, and $h_{0,0}(t)=\exp\left(0.14\times t\right)-0.5$. For crossover hazards (Panel G), we let $h_{1,1}(t)=2\times\exp\left(0.168\times t\right)-0.4$, $h_{1,0}(t)=1.5\times\exp\left(0.168\times t\right)-0.4$, $h_{0,1}(t)=\exp\left(0.168\times t\right)+0.7$, and $h_{0,0}(t)=0.5\times\exp\left(0.168\times t\right)+0.7$. Figure 3 is for the alternative hypothesis, that is, $\text{IC}(t)=h_{1,1}(t)-h_{1,0}(t)-h_{0,1}(t)+h_{0,0}(t)\neq0$ for some $t$. Here we replaced $h_{1,1}(t)$ of the above three scenarios under the null hypothesis with $h_{1,1}(t)=3\times\exp\left(0.14\times t\right)$ (Panel A), $h_{1,1}(t)=\exp\left(0.14\times t\right)+2$ (Panel D), and $h_{1,1}(t)=2.5\times\exp\left(0.182\times t\right)$ (Panel G), respectively. The simulation results are shown below:

1. Under the null hypothesis of no mechanistic interaction


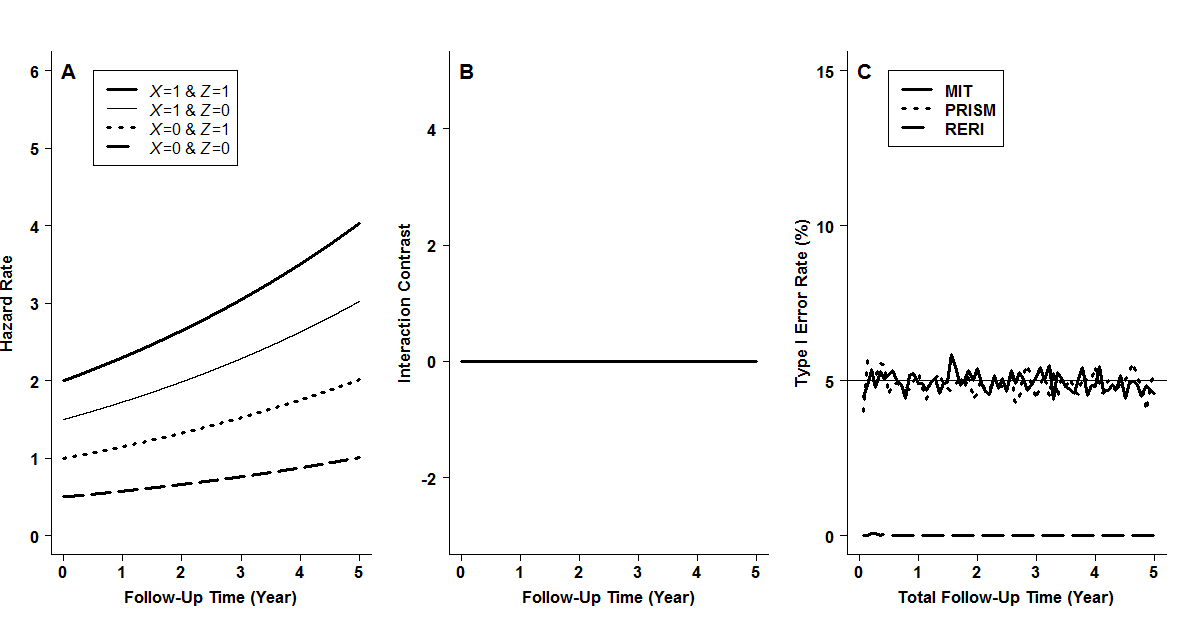


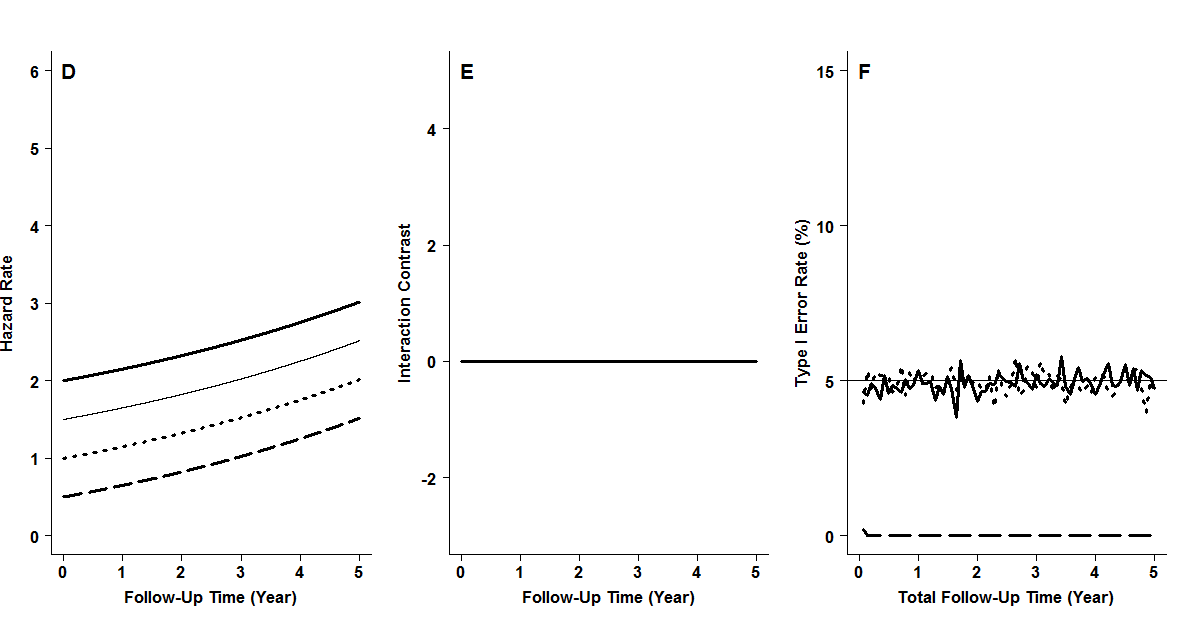


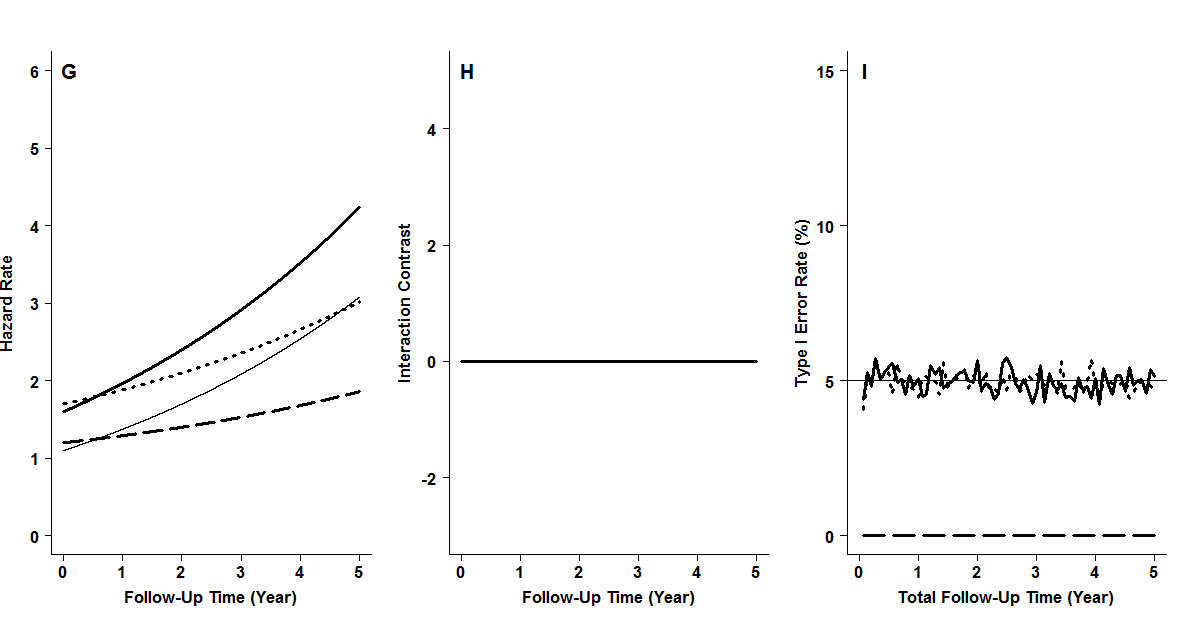


1. Under the alternative hypothesis of mechanistic interaction


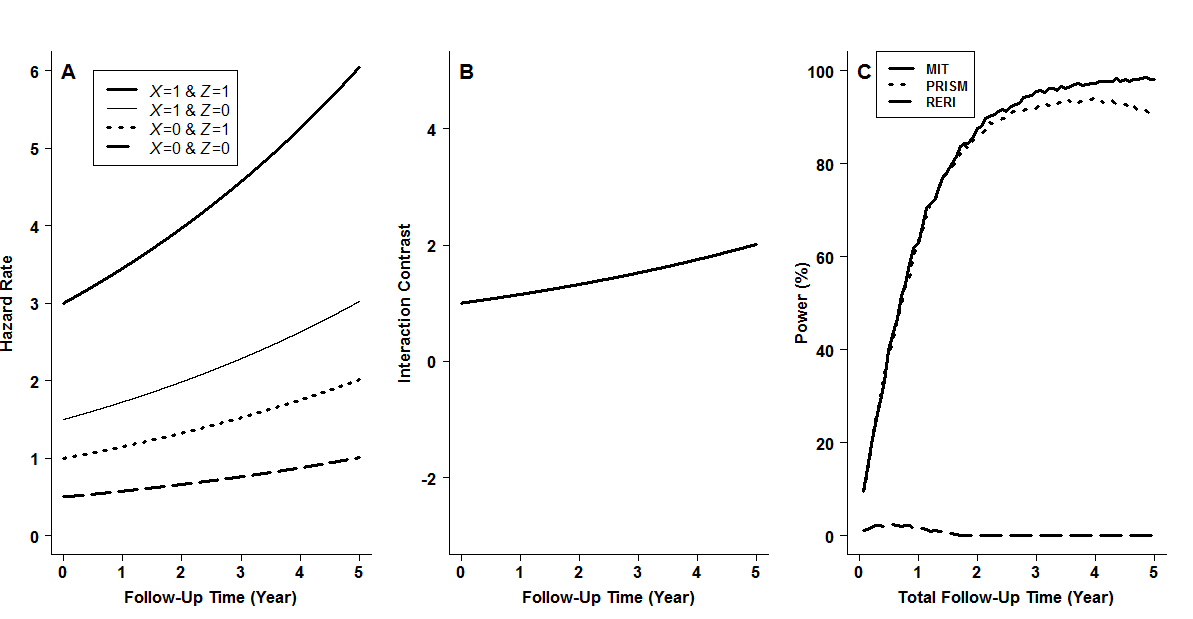


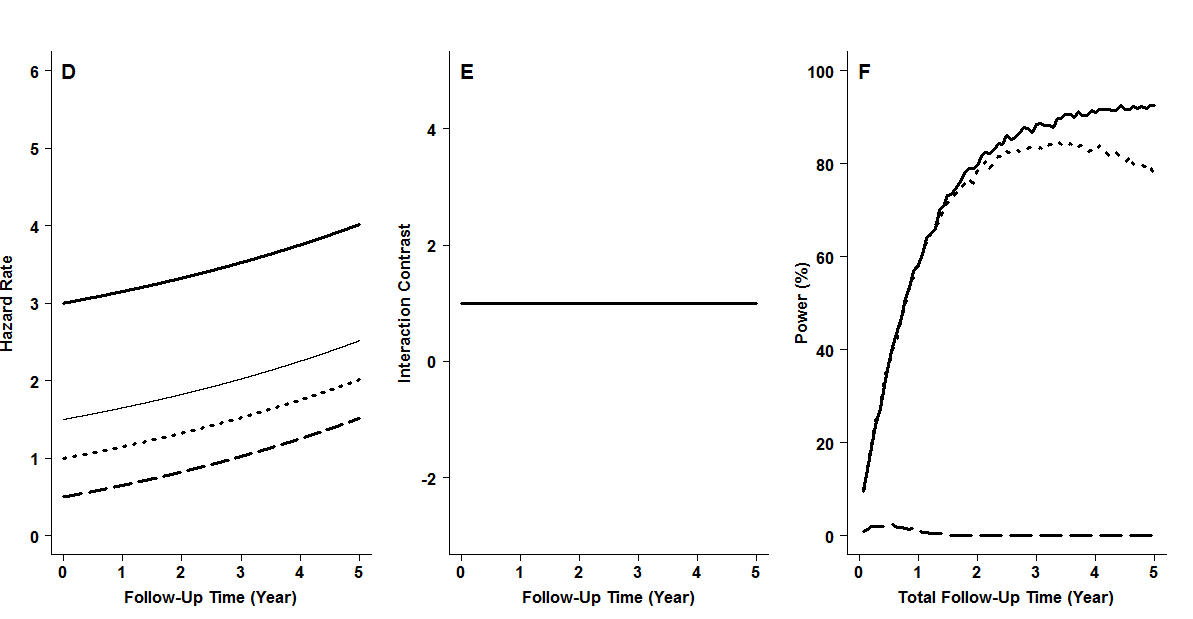


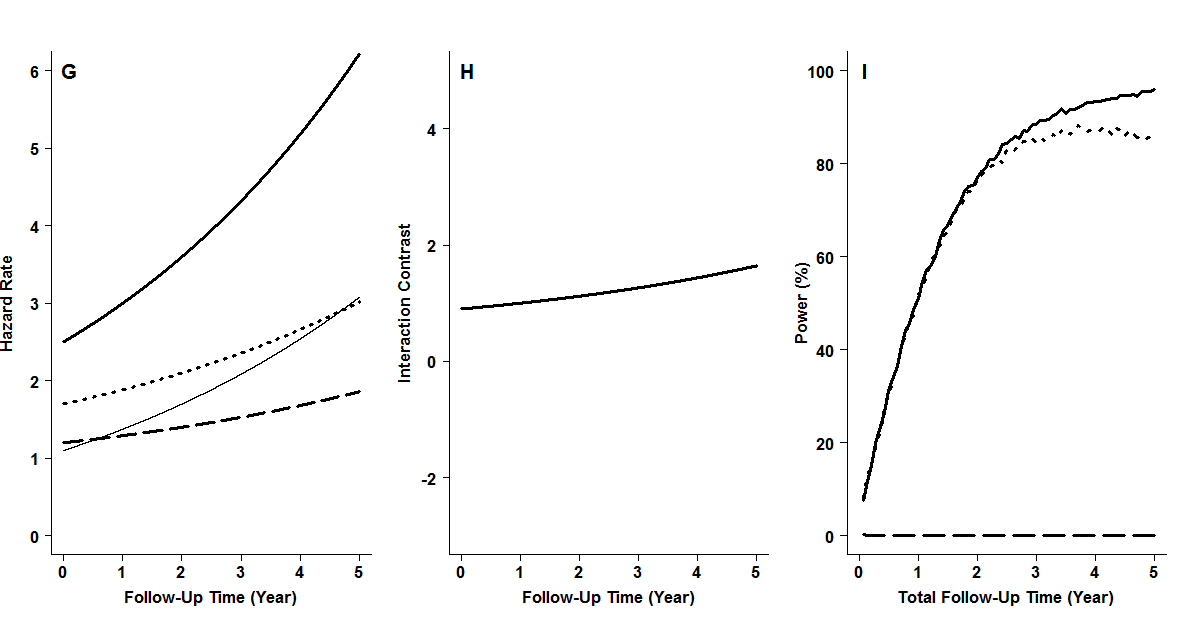

Supplement: S3 Appendix — (DOC) [file pone.0121638.s003.doc]
