# Supplementary material for: Testing for Mechanistic Interactions in Long-Term Follow-Up Studies
Source: PLoS One. 2015 Mar 26;10(3):e0121638. doi: 10.1371/journal.pone.0121638 (PMC4374952; doi:10.1371/journal.pone.0121638)
Supplement: S4 Appendix — (DOC) [file pone.0121638.s004.doc]

**S4 Appendix.**

The RERI and PRISM tests were modified for censored data scenarios in this paper. First, we use Kaplan-Meier method to estimate $\widehat{\text{S}}_{x,z}$, the cumulative survival proportion (probability) for people with exposure profile of $X=x,Z=z$ in certain time interval. For RERI, we test (one-sided) whether the index is statistically larger than one, or equivalently, $\widehat{\text{Risk}}_{1,1}-\widehat{\text{Risk}}_{1,0}-\widehat{\text{Risk}}_{0,1}=\left(1-\widehat{\text{S}}_{1,1}\right)-\left(1-\widehat{\text{S}}_{1,0}\right)-\left(1-\widehat{\text{S}}_{0,1}\right)>0$. We calculate its variance using Greenwood’s formula:

$$\begin{matrix} \text{Var}\left(\widehat{\text{Risk}}_{1,1}-\widehat{\text{Risk}}_{1,0}-\widehat{\text{Risk}}_{0,1}\right)=\text{Var}\left(\widehat{\text{S}}_{1,1}\right)+\text{Var}\left(\widehat{\text{S}}_{1,0}\right)+\text{Var}\left(\widehat{\text{S}}_{0,1}\right) \\ \text{ }=\widehat{\text{S}}_{1,1}^{2}\times\sum_{j=1}^{J} \frac{d_{11j}}{n_{11j}\times\left(n_{11j}-d_{11j}\right)} \\ \text{ }+\widehat{\text{S}}_{1,0}^{2}\times\sum_{j=1}^{J} \frac{d_{10j}}{n_{10j}\times\left(n_{10j}-d_{10j}\right)} \\ \text{ }+\widehat{\text{S}}_{0,1}^{2}\times\sum_{j=1}^{J} \frac{d_{01j}}{n_{01j}\times\left(n_{01j}-d_{01j}\right)}. \end{matrix}$$

The modified RERI test is a Z-test: $\text{Z=}\frac{\widehat{\text{Risk}}_{1,1}-\widehat{\text{Risk}}_{1,0}-\widehat{\text{Risk}}_{0,1}}{\sqrt{\text{Var}\left(\widehat{\text{Risk}}_{1,1}-\widehat{\text{Risk}}_{1,0}-\widehat{\text{Risk}}_{0,1}\right)}}.$ For PRISM, we test (two-sided) whether log PRISM is statistically different from zero. In a similar vein, an estimate of PRISM is $\widehat{\text{PRISM}}=\frac{\widehat{\text{S}}_{1,1}^{-1}\times\widehat{\text{S}}_{0,0}^{-1}}{\widehat{\text{S}}_{1,0}^{-1}\times\widehat{\text{S}}_{0,1}^{-1}}$ and the variance is

$$\begin{matrix} \text{Var}\left(\log\widehat{\text{PRISM}}\right)=\text{Var}\left[\log\left(\frac{\widehat{\text{S}}_{1,1}^{-1}\times\widehat{\text{S}}_{0,0}^{-1}}{\widehat{\text{S}}_{1,0}^{-1}\times\widehat{\text{S}}_{0,1}^{-1}}\right)\right] \\ \text{ }=\text{Var}\left(\log\widehat{\text{S}}_{1,1}\right)+\text{Var}\left(\log\widehat{\text{S}}_{1,0}\right) \\ \text{ +Var}\left(\log\widehat{\text{S}}_{0,1}\right)\text{+Var}\left(\log\widehat{\text{S}}_{0,0}\right) \\ \text{ }=\sum_{j=1}^{J} \frac{d_{11j}}{n_{11j}\times\left(n_{11j}-d_{11j}\right)}+\sum_{j=1}^{J} \frac{d_{10j}}{n_{10j}\times\left(n_{10j}-d_{10j}\right)} \\ \text{ +}\sum_{j=1}^{J} \frac{d_{01j}}{n_{01j}\times\left(n_{01j}-d_{01j}\right)}+\sum_{j=1}^{J} \frac{d_{00j}}{n_{00j}\times\left(n_{00j}-d_{00j}\right)}. \end{matrix}$$

The PRISM test is also a Z-test: $Z=\frac{\log\widehat{\text{PRISM}}}{\sqrt{\text{Var}(\log\widehat{\text{PRISM}})}}.$
