# Supplementary material for: Testing for Mechanistic Interactions in Long-Term Follow-Up Studies
Source: PLoS One. 2015 Mar 26;10(3):e0121638. doi: 10.1371/journal.pone.0121638 (PMC4374952; doi:10.1371/journal.pone.0121638)

**S5 Appendix.**

Here we let subjects with different exposure profiles to have different censoring rates: 0.8, 0.6, 0.4, and 0.2 , respectively for A, 0.6, 0.3, 0.2, and 0.1, respectively for B, and 0.4, 0.5, 0.2, and 0.1, respectively for C. The following figures show type I error rates for PRISM (left panels) and MIT (right panels):

1. Proportional hazards under the null hypothesis of no mechanistic interaction (cf. Panel A in Figure 2)


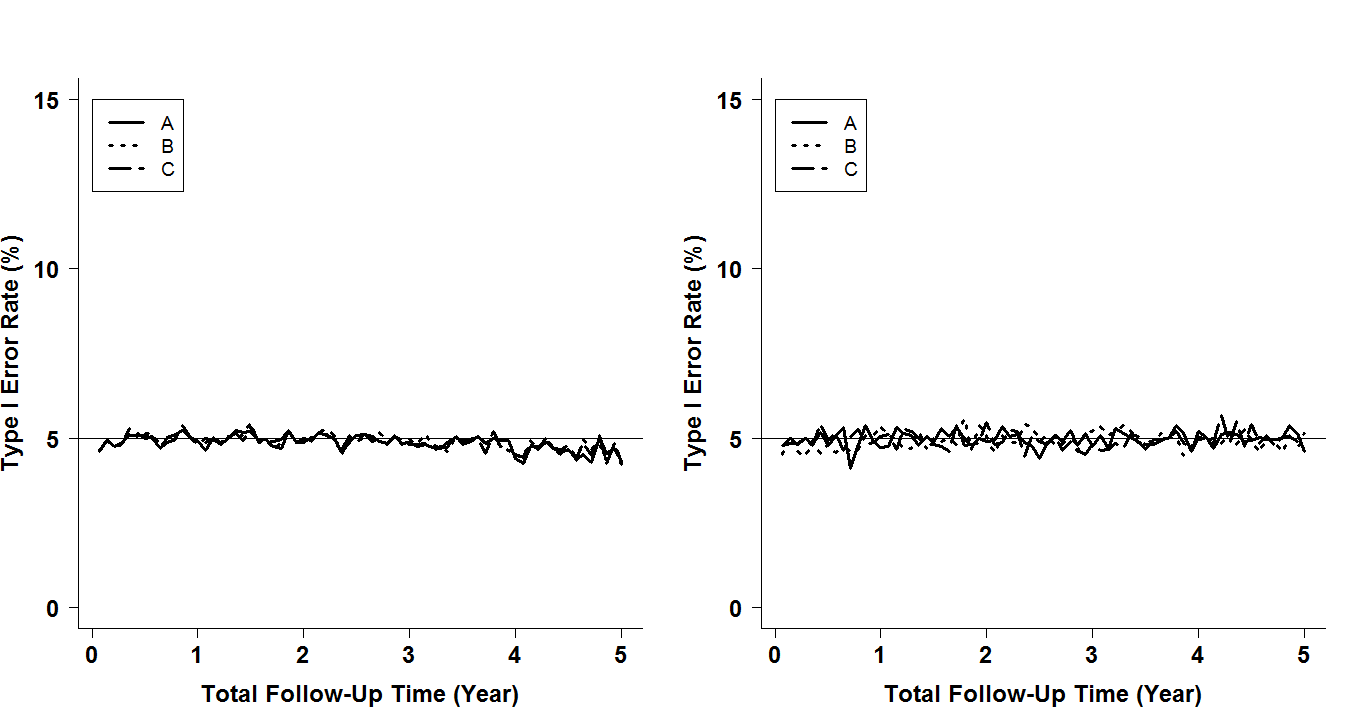


1. Non-proportional hazards under the null hypothesis of no mechanistic interaction (cf. Panel D in Figure 2)


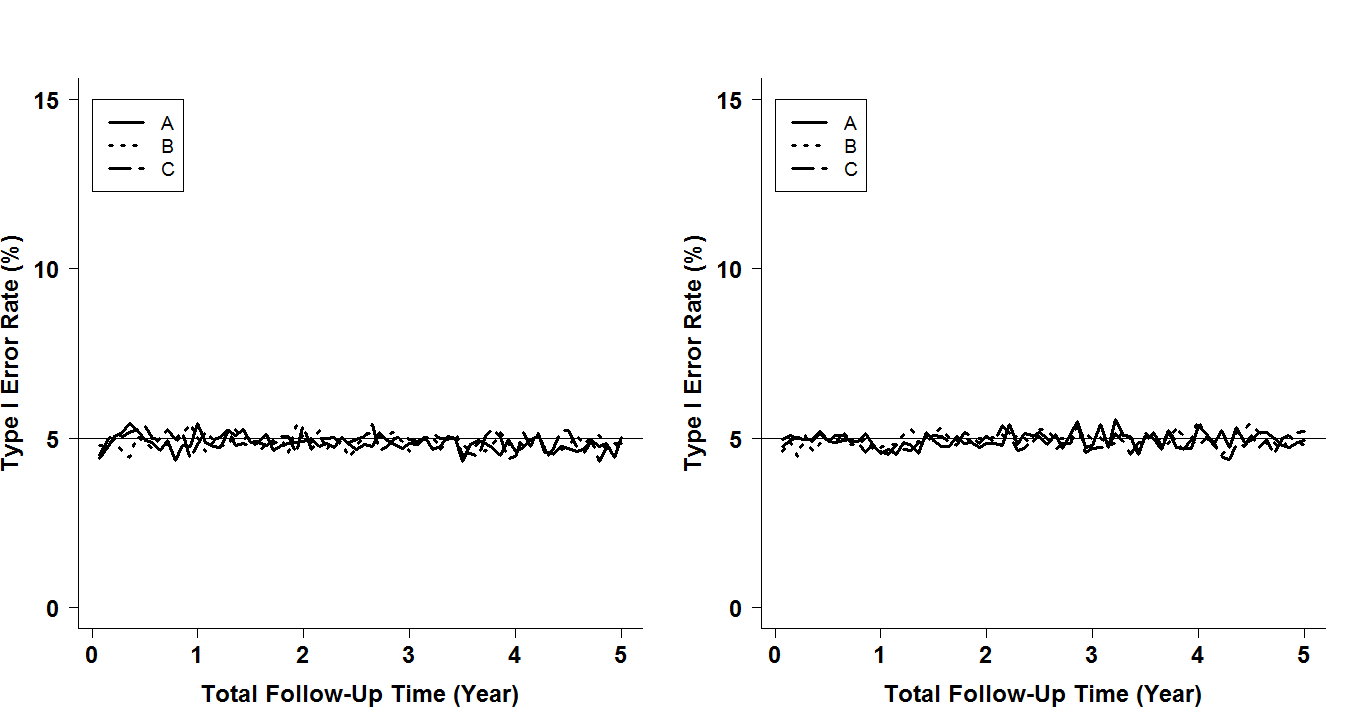


1. Crossover hazards under the null hypothesis of no mechanistic interaction (cf. Panel G in Figure 2)


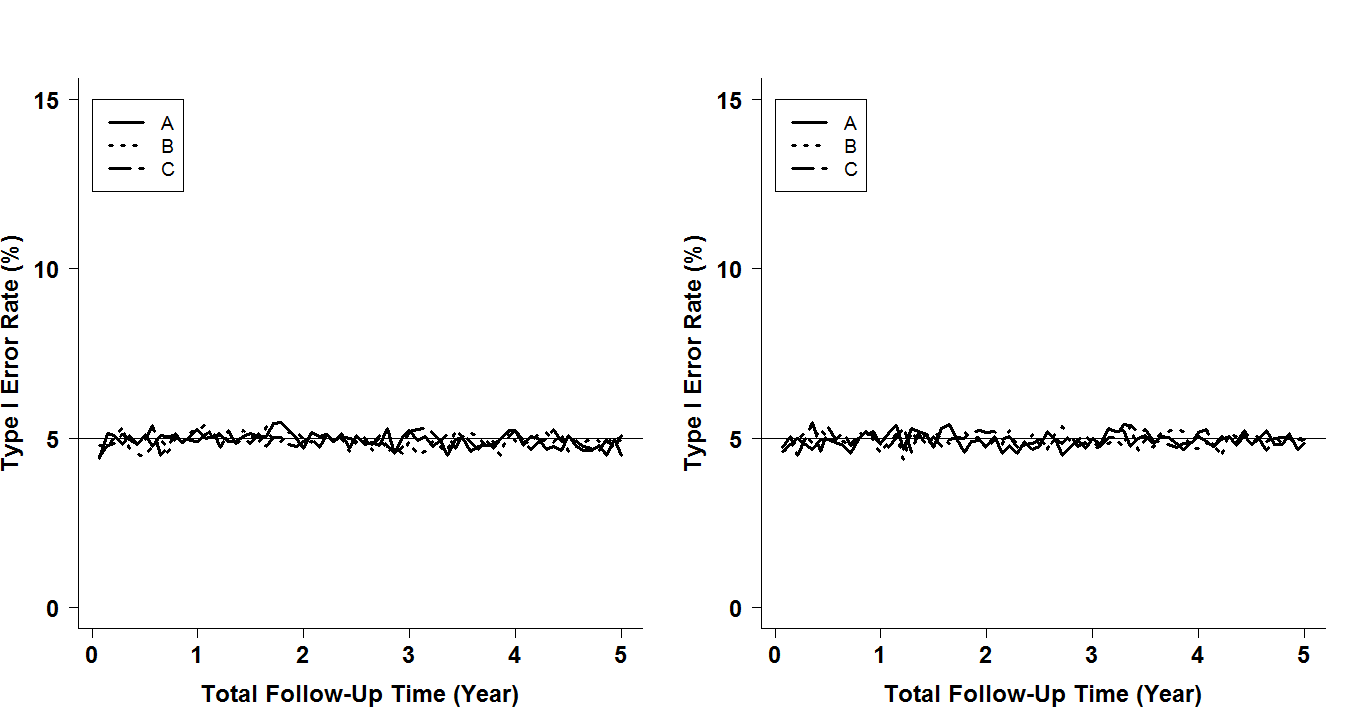

Supplement: S5 Appendix — (DOC) [file pone.0121638.s005.doc]
