# Supplementary material for: Testing for Mechanistic Interactions in Long-Term Follow-Up Studies
Source: PLoS One. 2015 Mar 26;10(3):e0121638. doi: 10.1371/journal.pone.0121638 (PMC4374952; doi:10.1371/journal.pone.0121638)

**S6 Appendix.**

Here we consider smaller sample sizes: 100 subjects (in each exposure profile) for A, 75 subjects for B, and 50 subjects for C. The following figures (I) ~ (III) show type I error rates for PRISM (left panels) and MIT (right panels). The following figures (IV) ~ (VI) show powers for PRISM (left panels) and MIT (right panels).

1. Proportional hazards under the null hypothesis of no mechanistic interaction (cf. Panel A in Figure 2)


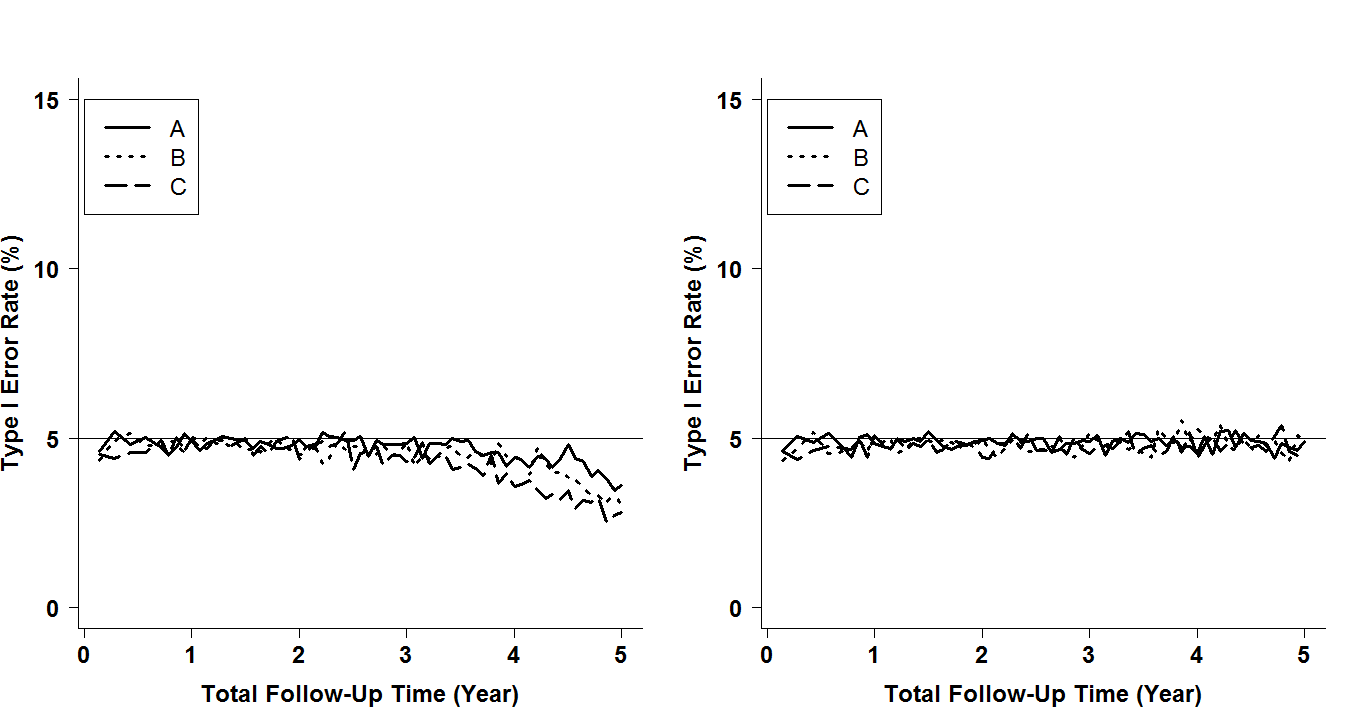


1. Non-proportional hazards under the null hypothesis of no mechanistic interaction (cf. Panel D in Figure 2)


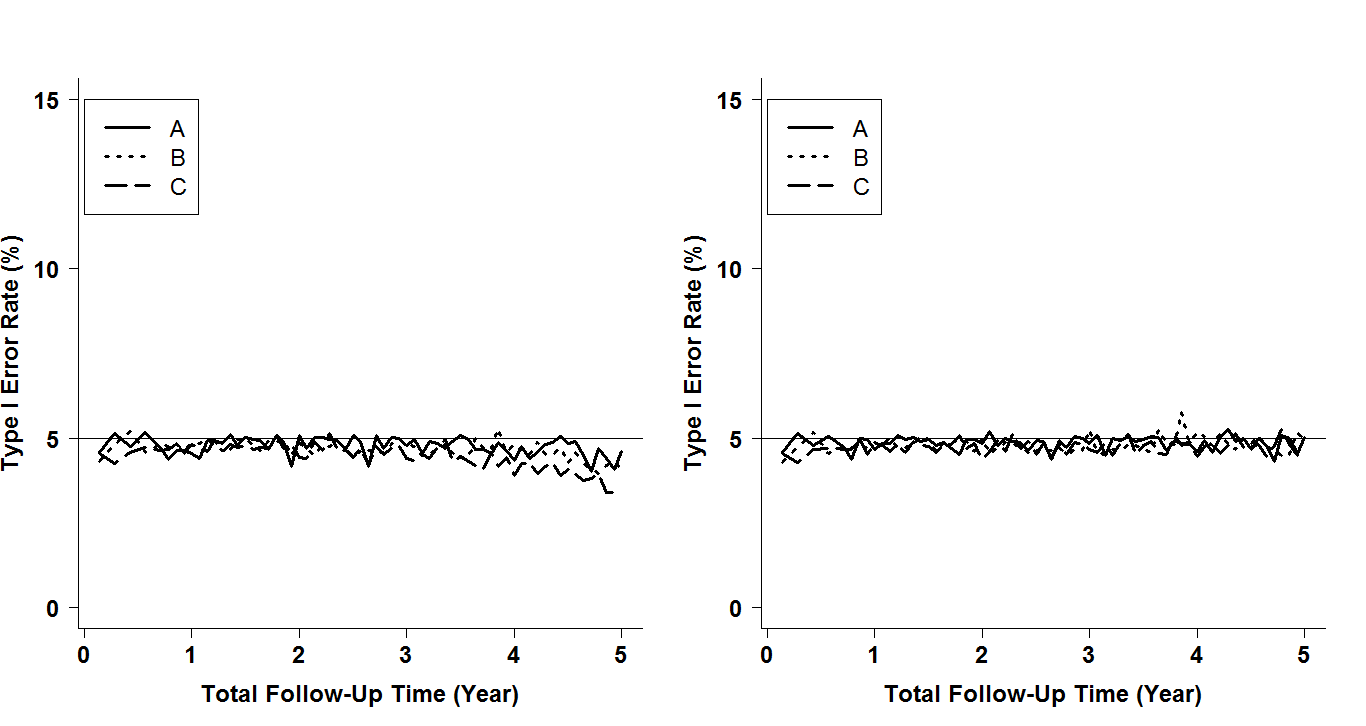


1. Crossover hazards under the null hypothesis of no mechanistic interaction (cf. Panel G in Figure 2)


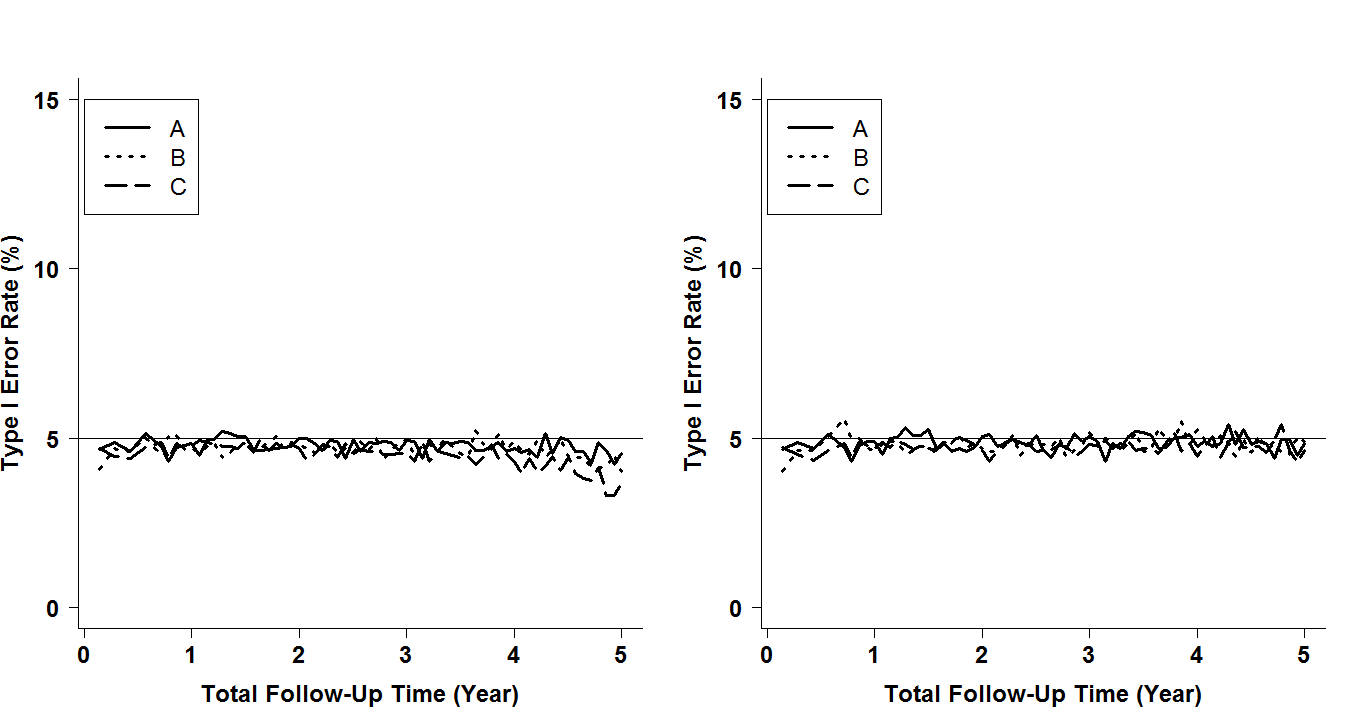


1. Proportional hazards under the alternative hypothesis of no mechanistic interaction (cf. Panel A in Figure 3)


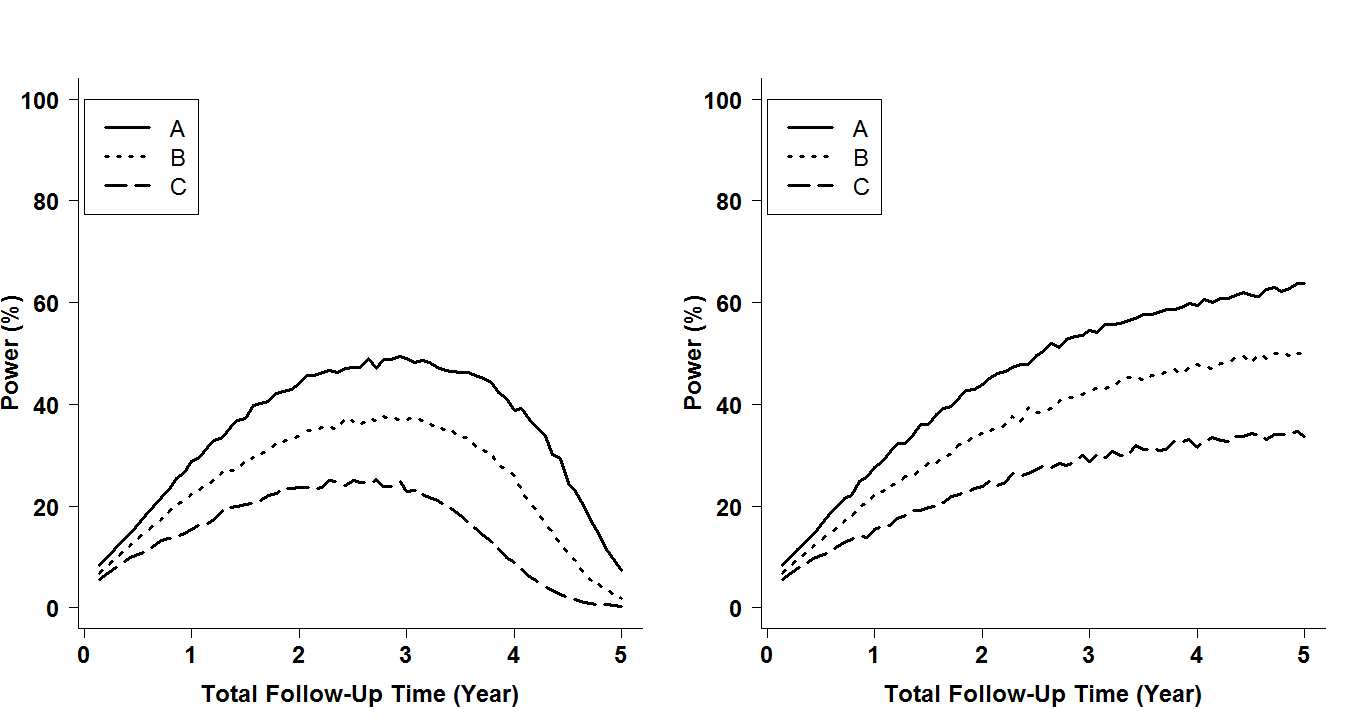


1. Non-proportional hazards under the alternative hypothesis of no mechanistic interaction (cf. Panel D in Figure 3)


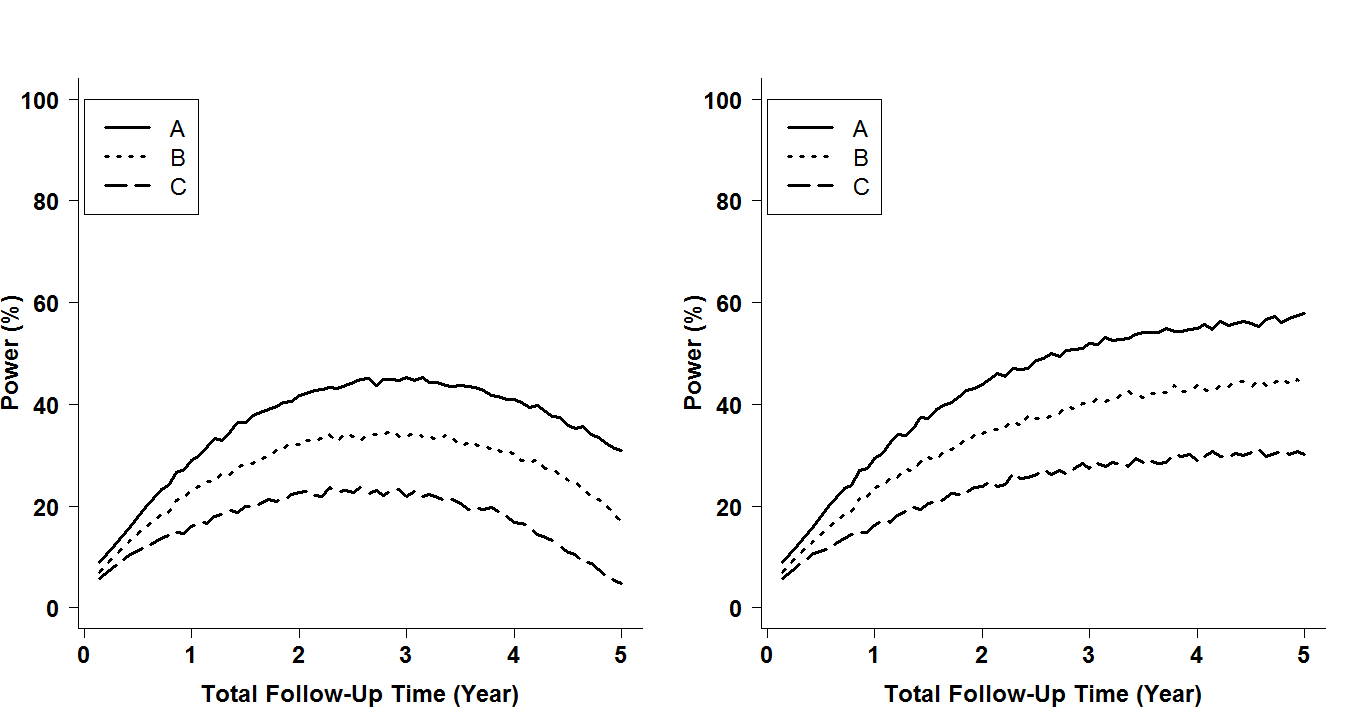


1. Crossover hazards under the alternative hypothesis of no mechanistic interaction (cf. Panel G in Figure 3)


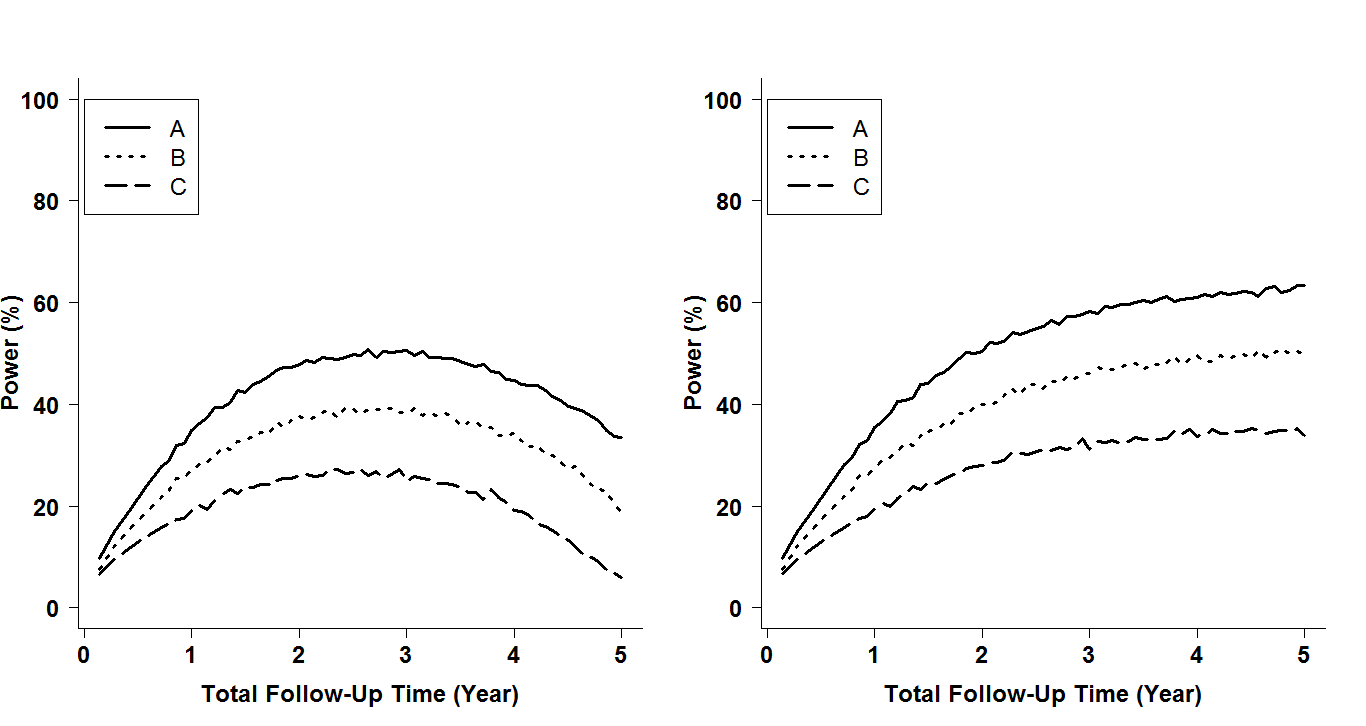

Supplement: S6 Appendix — (DOC) [file pone.0121638.s006.doc]
