# Supplementary material for: Testing for Mechanistic Interactions in Long-Term Follow-Up Studies
Source: PLoS One. 2015 Mar 26;10(3):e0121638. doi: 10.1371/journal.pone.0121638 (PMC4374952; doi:10.1371/journal.pone.0121638)

**S8 Appendix.**

Here we present additionally the results of the RERI test under the assumption of monotonicity (testing for $\text{RERI}>0$). The following figures show type I error rates (left panels) and empirical powers (right panels):

1. Proportional hazards (cf. Panel A in Figures 2 and 3)


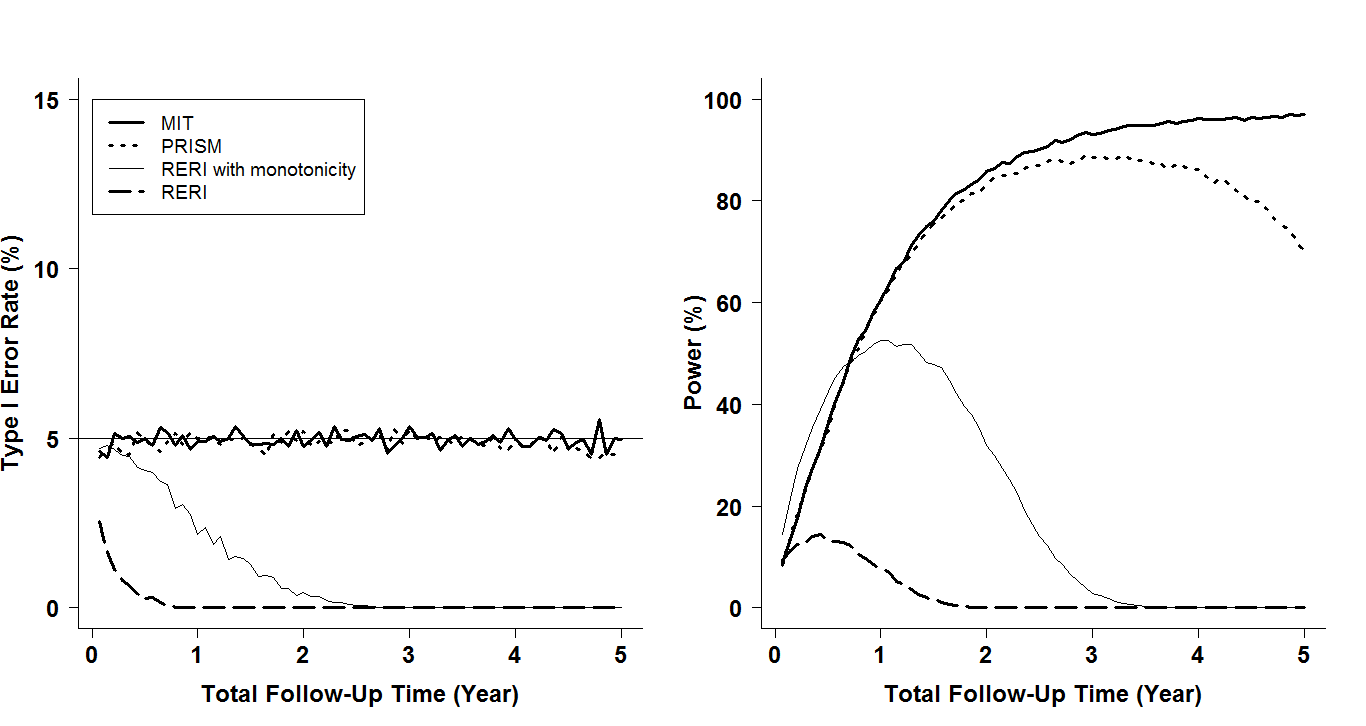


1. Non-proportional hazards (cf. Panel D in Figures 2 and 3)


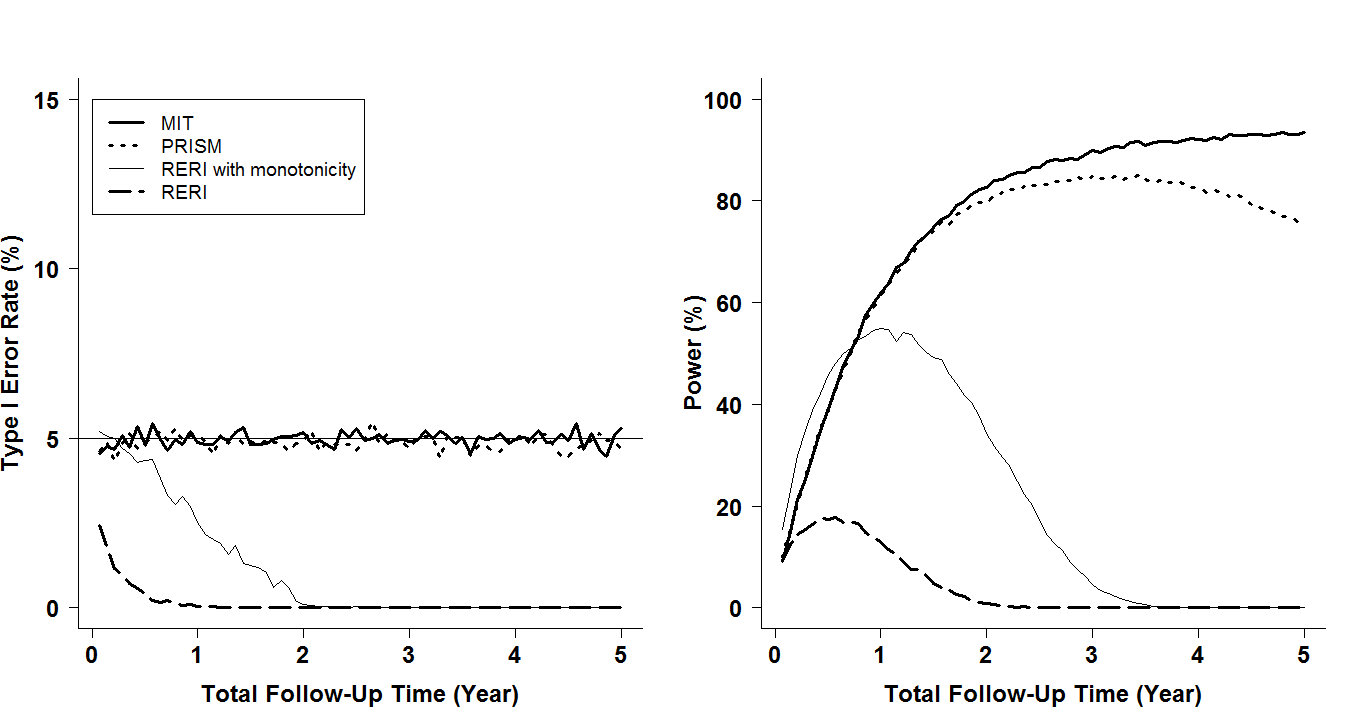


1. Crossover hazards (cf. Panel G in Figures 2 and 3)


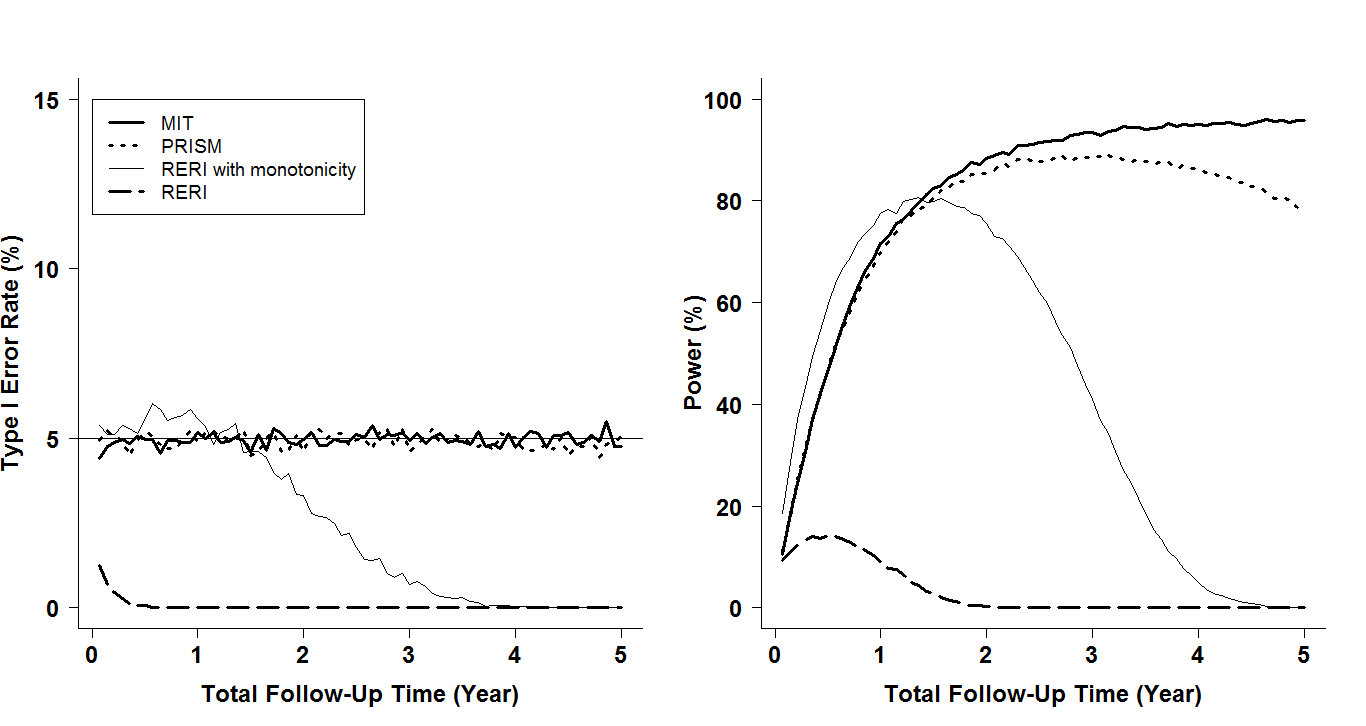

Supplement: S8 Appendix — (DOC) [file pone.0121638.s008.doc]
