# Supplementary material for: Testing for Mechanistic Interactions in Long-Term Follow-Up Studies
Source: PLoS One. 2015 Mar 26;10(3):e0121638. doi: 10.1371/journal.pone.0121638 (PMC4374952; doi:10.1371/journal.pone.0121638)
Supplement: S9 Appendix — (DOC) [file pone.0121638.s009.doc]

**S9 Appendix.**

We first use the Nelson-Aalen method to estimate the cumulative hazards (CH’s) and calculate the logarithm of PRISM:

$$\begin{matrix} \log\widehat{\text{PRISM}}=\widehat{\text{CH}}_{1,1}-\widehat{\text{CH}}_{1,0}-\widehat{\text{CH}}_{0,1}+\widehat{\text{CH}}_{0,0} \\ \text{ }=\sum_{j=1}^{J} \frac{d_{11j}}{n_{11j}}-\sum_{j=1}^{J} \frac{d_{10j}}{n_{10j}}-\sum_{j=1}^{J} \frac{d_{01j}}{n_{01j}}+\sum_{j=1}^{J} \frac{d_{00j}}{n_{00j}} \\ \text{ }=\sum_{j=1}^{J} \left(\frac{d_{11j}}{n_{11j}}-\frac{d_{10j}}{n_{10j}}-\frac{d_{01j}}{n_{01j}}+\frac{d_{00j}}{n_{00j}}\right) \\ \text{ }=\sum_{j=1}^{J} \text{IC}_{j}. \end{matrix}$$

Next, we turn to Greenwood’s method to estimate the variances of survival proportions (S’s) and then calculate the variance of logarithm of PRISM:

$$\begin{matrix} \text{Var}(\log\widehat{\text{PRISM}})=\text{Var}\left[\log\left(\frac{\widehat{\text{S}}_{1,1}^{-1}\times\widehat{\text{S}}_{0,0}^{-1}}{\widehat{\text{S}}_{1,0}^{-1}\times\widehat{\text{S}}_{0,1}^{-1}}\right)\right] \\ \text{ }=\text{Var}\left(\log\widehat{\text{S}}_{1,1}\right)+\text{Var}\left(\log\widehat{\text{S}}_{1,0}\right) \\ \text{ +Var}\left(\log\widehat{\text{S}}_{0,1}\right)\text{+Var}\left(\log\widehat{\text{S}}_{0,0}\right) \\ \text{ }=\sum_{j=1}^{J} \frac{d_{11j}}{n_{11j}\times\left(n_{11j}-d_{11j}\right)}+\sum_{j=1}^{J} \frac{d_{10j}}{n_{10j}\times\left(n_{10j}-d_{10j}\right)} \\ \text{ +}\sum_{j=1}^{J} \frac{d_{01j}}{n_{01j}\times\left(n_{01j}-d_{01j}\right)}+\sum_{j=1}^{J} \frac{d_{00j}}{n_{00j}\times\left(n_{00j}-d_{00j}\right)} \\ \text{ }\approx\sum_{j=1}^{J} \frac{d_{11j}}{n_{11j}^{2}}+\sum_{j=1}^{J} \frac{d_{10j}}{n_{10j}^{2}}+\sum_{j=1}^{J} \frac{d_{01j}}{n_{01j}^{2}}+\sum_{j=1}^{J} \frac{d_{00j}}{n_{00j}^{2}} \\ \text{ }=\sum_{j=1}^{J} \left(\frac{d_{11j}}{n_{11j}}-\frac{d_{10j}}{n_{10j}}-\frac{d_{01j}}{n_{01j}}+\frac{d_{00j}}{n_{00j}}\right)^{2} \\ \text{ }=\sum_{j=1}^{J} \text{IC}_{j}^{2}, \end{matrix}$$

where we assumed that there is at most one subject contracting the disease at a risk set such that for each $j$ we have $d_{11j}^{2}=d_{11j}$ and $d_{11j}\times d_{10j}=d_{11j}\times d_{01j}=d_{11j}\times d_{00j}=0$ for exposure profile 1,1 and likewise for the other three exposure profiles. The PRISM test is therefore

$$Z=\frac{\log\widehat{\text{PRISM}}}{\sqrt{\text{Var}(\log\widehat{\text{PRISM}})}}\approx\frac{\sum_{j=1}^{J} \text{IC}_{j}}{\sqrt{\sum_{j=1}^{J} \text{IC}_{j}^{2}}}.$$

We immediately recognize that it is a hazard-rate additivity test with (approximately) equal weight attached to the risk sets.
